# Modelled Response Times for Police and Fire Brigade Co-Dispatch to Out-of-Hospital Cardiac Arrest: A Geospatial Simulation Study in Vorarlberg, Austria

**DOI:** 10.3390/jcm15218237

**Published:** 2026-10-22

**Authors:** Andreas Schmid, Harald Rinösl, Manuel Winkler, Maximilian Scheidl, Oliver Kimberger, Mathias Maleczek

**Affiliations:** 1 Clinical Division of Anesthesiology and Intensive Care Medicine, Academic Teaching Hospital Feldkirch, 6800 Vorarlberg, Austria; 2 Clinical Division of General Anaesthesia and Intensive Care Medicine, Department of Anaesthesia, Intensive Care Medicine and Pain Medicine, Medical University of Vienna, 1090 Vienna, Austria; 3 Ludwig Boltzmann Institute Digital Health and Patient Safety, 1090 Vienna, Austria

**Keywords:** out-of-hospital cardiac arrest, cardiopulmonary resuscitation, first responder, co-dispatch, response time, no-flow time, emergency medical services, police, fire brigade, geospatial simulation

## Abstract

**Background/Objectives:** Survival after out-of-hospital cardiac arrest (OHCA) depends on the interval between collapse and the start of chest compressions. We estimated how co-dispatching police and volunteer fire brigades alongside emergency medical services (EMS) would change the modelled time from dispatch to arrival of the first unit in Vorarlberg, Austria. **Methods:** In a geospatial simulation, 500 OHCA locations were drawn from a 100 m population raster, weighted by residential population. Driving times from all 18 EMS, 28 police and 121 volunteer fire stations were routed on the road network, and turnout times of 2 min (EMS), 0 min (police) and 5 min (fire brigade) were added; all units were modelled as continuously available at fixed stations. Four configurations (EMS alone, EMS plus police, EMS plus fire brigade, triple dispatch) were compared by paired time gains and 10 min coverage with bootstrap confidence intervals (CIs); uncertainty from sampling, turnout times, unit availability and routing was assessed separately. **Results:** Of 500 missions, 499 were analysable. Median response time was 9.81 min with EMS alone, 5.45 min with EMS plus police, 7.36 min with EMS plus fire brigade and 5.41 min with triple dispatch; the paired median time gain of triple dispatch over EMS alone was 4.34 min (95% CI 3.80–4.67). Locations reached within 10 min rose from 51.1% to 87.4%, 87.0% and 94.6%, and the resident population inside the 10 min isochrones rose from 55.6% to 90.9%, 91.9% and 95.5%. **Conclusions:** Under fixed station locations, continuous unit availability and assumed agency-specific turnout times, police and fire brigade co-dispatch increased modelled ten-minute coverage compared with EMS alone. Police arrived earlier at most locations, whereas the denser fire station network contributed most in rural areas; their combination reached the most locations. Operational validation is required before these estimates can inform regional implementation.

## 1. Introduction

Out-of-hospital cardiac arrest (OHCA) represents a major public health challenge, characterised by both high incidence and low survival rates [1,2,3]. Despite substantial advancements in emergency medical services (EMS), survival rates often depend heavily on the time elapsed between collapse and the initiation of cardiopulmonary resuscitation (CPR). The International Liaison Committee on Resuscitation (ILCOR) and the European Resuscitation Council (ERC) consistently emphasise that the chain of survival hinges on early recognition and immediate bystander or first-responder chest compressions [4,5,6]. In the first minutes after cardiac arrest, minimising the no-flow interval is paramount for preserving neurological function and achieving return of spontaneous circulation (ROSC); in a model derived from witnessed out-of-hospital cardiac arrests with ventricular fibrillation, Larsen et al. estimated that survival decreased by 2.3 percentage points per minute of delay to CPR, by 1.1 percentage points per minute of delay to defibrillation and by 2.1 percentage points per minute of delay to advanced life support, as absolute decreases from an estimated survival of 67% with immediate treatment. These historical estimates refer to this selected population and should not be read as a universal survival decrement for all OHCA [7,8,9]. Bystander CPR is therefore vital, yet high bystander CPR rates remain difficult to achieve despite many educational and organisational efforts [10]. Bibliometric analysis has identified out-of-hospital cardiac arrest and simulation among the emerging themes in cardiopulmonary resuscitation research [11].

**Figure 1:**
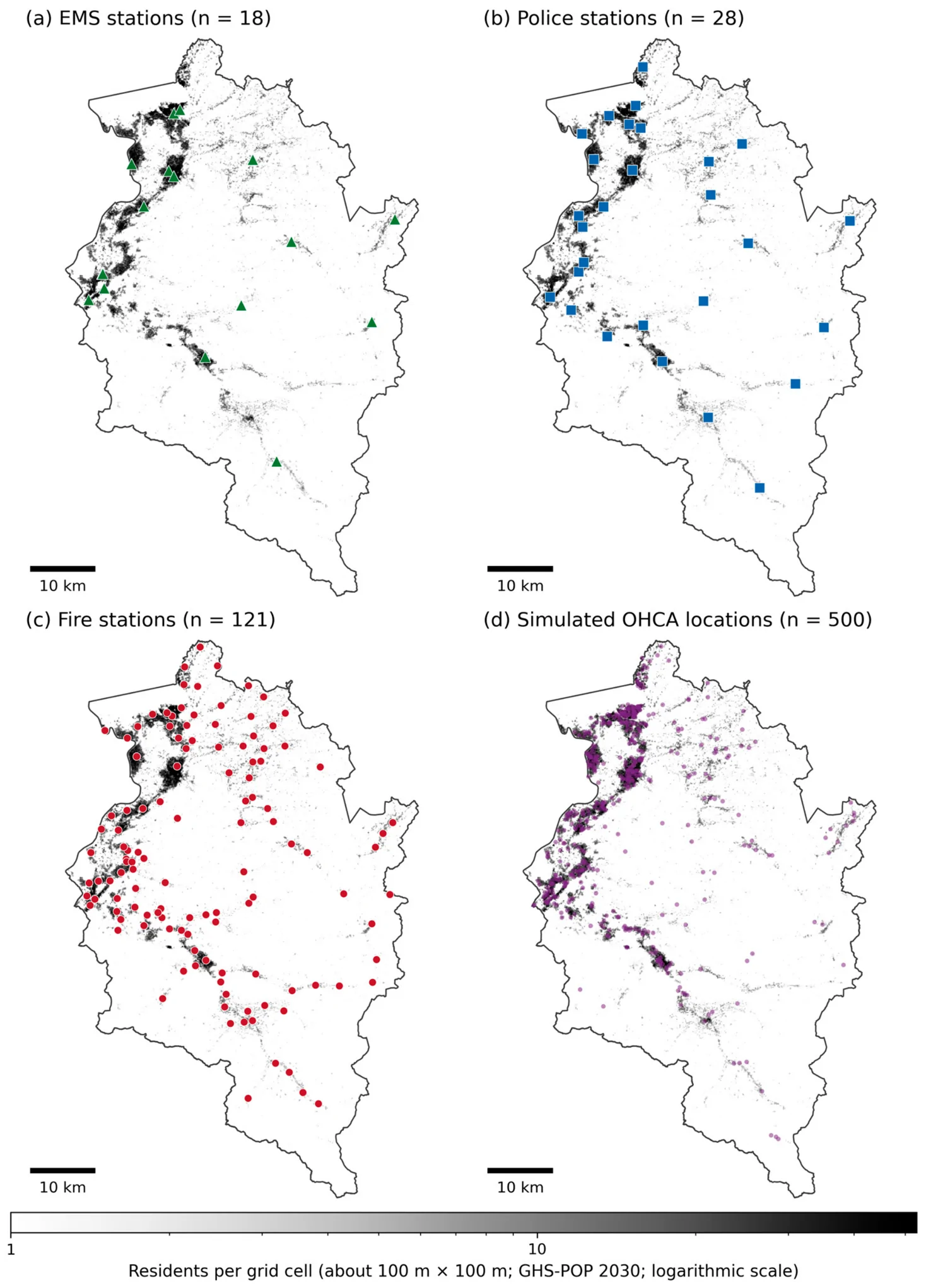
Distribution of responder stations and simulated incident locations across Vorarlberg, overlaid on the 100 m residential population grid. (**a**) EMS stations (*n* = 18, including four physician-staffed response car bases); (**b**) police stations (*n* = 28); (**c**) volunteer fire stations (*n* = 121); (**d**) the 500 simulated out-of-hospital cardiac arrest locations sampled with probability proportional to population. Grey shading: residents per grid cell (GHS-POP, epoch 2030; logarithmic scale), as shown in the legend below the panels.

In the federal state Vorarlberg, Austria, the regional epidemiology of OHCA presents particular structural and geographical challenges. While the densely populated Rhine Valley allows rapid EMS response capabilities, the alpine topography of side valleys such as the Montafon or Bregenzerwald introduces inherent transit delays. Austrian logistics operate under a fixed-station paradigm in which professional and volunteer emergency medical technicians and emergency doctors are dispatched from a limited number of centralised bases; the distribution of EMS, fire and police stations are displayed in Figure 1. Reaching a patient early is therefore structurally difficult in peripheral zones, which highlights the need for dispatch strategies that leverage decentralised civil resources. According to the regional data of the German Resuscitation Registry (Academic Teaching Hospital Feldkirch, 2024, unpublished); all cardiac arrests in Vorarlberg treated by the emergency medical services, excluding patients treated by helicopter emergency physicians), the incidence of OHCA in Vorarlberg was 127.2 per 100,000 inhabitants in 2024, and 71.9 per 100,000 received a resuscitation attempt (299 cases); the first vehicle, including first responders, arrived within 8 min of the alarm in 73.2% of resuscitations, and telephone-assisted CPR (T-CPR) was provided in 24.1% of all cardiac arrests (72 of 299; status unknown in 18).

Internationally, alternative first responders such as police officers or firefighters are used to bridge the interval before ambulance arrival. Successful programmes have been reported from North Carolina, Oregon and Vienna, where police and fire units are dispatched simultaneously to OHCA in order to start high-quality chest compressions and early defibrillation [12,13]. Smartphone-based alerting of trained lay responders is a further option to shorten the no-flow interval [14,15]. While metropolitan data confirms the benefits of dual and triple dispatch of municipal services, the efficacy of scaling such framework across a heterogenous region such as Vorarlberg remains unclear. A large registry study found higher ROSC and survival rates after OHCA in European countries with functioning first responder systems [15,16].

In a previous simulation study in Lower Austria, our group showed that dual dispatch of volunteer fire services alongside EMS reduced mean response time from 10.6 to 7.2 min and raised the proportion of events reached within 10 min from 49.0% to 92.6% [17]. That analysis covered a single additional agency in a predominantly lowland region served by 1590 fire stations. Whether the same holds in a smaller, topographically constrained state, and whether police units, which unlike volunteer firefighters do not first have to travel to a station, add further benefit, has not been examined. The present study extends the approach to a three-agency comparison in Vorarlberg, samples incident locations weighted by residential population, and complements the mission-based estimates with an isochrone-based coverage analysis. For comparability with that earlier work, the turnout times assumed for EMS and for the volunteer fire brigades were deliberately kept identical. The primary endpoint was the modelled interval from dispatch to arrival of the first unit at a simulated incident coordinate. We used 10 min as a planning threshold for coverage, in line with response-time targets commonly used in EMS planning, and not as a clinical intervention window; results for other thresholds can be read from the cumulative distributions.

## 2. Materials and Methods

### 2.1. Study Design

A geospatial simulation study was conducted to model response times to resuscitation events. Because no patient-identifiable or real-world clinical data were used, no Ethics Committee vote was necessary following local legislation. The primary endpoint was the modelled interval from dispatch to arrival of the first unit at a simulated incident coordinate, compared between EMS alone and dual or triple dispatch of EMS, police and fire brigades and summarised as the paired time gain over EMS alone. Secondary endpoints were the proportion of locations reached within 10 min (≤10 min), the proportion of locations at which a non-EMS unit arrived first, and the area and resident population within 10 min drive-time isochrones.

The study was parameterised using spatial and demographic data from Vorarlberg, Austria’s westernmost federal state (2601 km^2^) [18]. Vorarlberg has a heterogeneous topography, combining the densely populated Rhine Valley and Walgau conurbations with rugged alpine regions and isolated valleys such as the Bregenzerwald and the Montafon. The state has approximately 412,000 residents, concentrated primarily around the three urban centres with more than 30,000 inhabitants (Dornbirn, Feldkirch and the capital Bregenz) [19]. While the urbanised valley corridors benefit from well-connected road networks and shorter access routes, the surrounding alpine terrain presents inherent operational challenges for EMS through extended travel distances and geographical barriers.

### 2.2. Emergency Medical Services

EMS in Vorarlberg are provided by the Austrian Red Cross, the Samariterbund and the Walser Rettung. The Rettungs- und Feuerwehrleitstelle Vorarlberg (RFL, Rotes Kreuz Vorarlberg), the dispatch centre for EMS and fire brigades, provided the geolocations of all stations. Thirteen Red Cross ambulance stations entered the simulation, ten of them staffed around the clock and three during daytime only, together with the station of the Walser Rettung in the Kleinwalsertal and four of the six bases for physician-staffed emergency response cars (Notarzteinsatzfahrzeug, NEF); the NEF bases in Bludenz and in the Bregenzerwald and the Samariterbund Feldkirch were merged with the co-located ambulance stations because of their immediate geographical proximity. This gave 18 EMS station locations in total (station inventory in Table S3); the Red Cross station and the NEF base in Hohenems, 28 m apart, were kept as separate units. Daytime-only stations were treated as continuously available, an assumption that favours the EMS-only configuration and therefore works against the effect reported here. Helicopter emergency medical services (HEMS) were not modelled. All ambulances carry a defibrillator, and NEF crews provide advanced life support. Turnout time for EMS was set to 2 min, which corresponds to the mean overall turnout time reported for EMS in Lower Austria [17] and reflects clinical practice; the official requirement in Vorarlberg is 1 min during daytime and 2 min at night. A turnout time of 1 min was examined in the sensitivity analysis.

### 2.3. Fire Brigade

Fire brigades in Vorarlberg consist almost exclusively of volunteers. Active members undergo standardised training in firefighting and technical rescue, including rudimentary first aid and basic life support (BLS) education. The official station register lists 144 fire stations, of which 23 are fire brigades serving a single factory or enterprise (data provided by the Federal Fire Brigade Association Vorarlberg, Austria). Those are not part of public emergency response and were excluded, leaving 121 volunteer fire stations for the simulation. In total, 7076 active members were registered. At the time of the study, fire units were not dispatched to OHCA. No consolidated official data on turnout times of Austrian volunteer fire brigades are available. Because volunteer members must travel from their home to the fire station upon alerting, the turnout time was standardised at 5 min, based on operational experience and on published data on determinants of turnout times in volunteer fire brigades [20]. Fire brigade members are trained in BLS and can start chest compressions on arrival; AED availability on fire vehicles is neither mandatory nor standardised.

### 2.4. Police

Police stations are distributed comparatively evenly across the rural and mountainous parts of the state and therefore provide broader geographical coverage than ambulance stations. There are 28 police stations with approximately 900 officers in executive service [21]. All police cars are equipped with automated external defibrillators (AEDs), and new police personnel undergo mandatory training in tactical medicine and basic life support. Police units were assumed to be dispatchable without delay at any time of day, and the turnout time was therefore set to 0 min; patrol movements were not modelled, and each unit was routed from its station. Station coordinates were checked for duplicates and verified against the official addresses of the Vorarlberg State Police Directorate (Table S3). The police station inventory corresponds to the 28 inspections reported by the Federal Ministry of the Interior; two further sites listed by the State Police Directorate (Satteins, Lochau) were not included because they are staffed only during the winter season. The locations of all EMS, fire and police stations are shown in Figure 1.

### 2.5. Geographical Data

Administrative boundaries of the four political districts of Vorarlberg (Bludenz, Bregenz, Dornbirn and Feldkirch) were taken from the official district shapefile of Statistik Austria (STATISTIK_AUSTRIA_POLBEZ, version 1 January 2025). Residential population was obtained from the Global Human Settlement Population Grid (GHS-POP, release R2023A, epoch 2030; raster GHS_POP_E2030_GLOBE_R2023A_54009_100_V1_0, tile R4_C19), a globally consistent raster of residential population at 100 m spatial resolution in the World Mollweide projection (ESRI:54009) [22,23]. The raster was clipped to the state boundary and cells with a population count of zero or less were masked. No scaling was applied. The resulting population within the study area was 394,736 residents, compared with an official population of 412,057 in 2025 [19]. In GHS-POP R2023A, the 2025 and 2030 epochs are model-based projections, whereas the most recent epoch not based on projection (2020) already lies several years in the past; the 2030 epoch was therefore chosen so that the model remains applicable over the planning horizon of the coming years, and the 2020 and 2025 epochs, which share the same grid, were used in a sensitivity analysis. The degree of urbanisation of each location was taken from the GHS settlement model grid (GHS-SMOD, epoch 2020) [24].

### 2.6. Generation of Simulated OHCA Locations

Because the incidence of OHCA is, to a first approximation, proportional to the resident population, 500 hypothetical OHCA locations were drawn from the masked population raster with replacement, the probability of selecting a given cell being proportional to its population count. The 500 locations of the original analysis were retained as the main sample. To quantify Monte Carlo uncertainty, 20 further independent samples of 500 locations were drawn with documented random seeds (20261014–20261033). This reproduces the settlement pattern of the state, including the concentration of population in the Rhine Valley and the sparse population of the alpine valleys, without requiring any patient-level data. The centre coordinate of each selected raster cell was transformed to WGS 84 and used as the incident location (Figure 1). Neither the time of day nor the day of week or season was modelled, and no assumptions were made regarding patient characteristics, witnessed status or initial rhythm.

### 2.7. Response Time Modelling

For every incident location, the driving time from every station of every agency to the incident was queried from the OpenRouteService (ORS 10.1) matrix endpoint using the driving-car profile on OpenStreetMap road data [25]. The shortest of these driving times was taken as the travel time of that agency, and the distance by which each location was snapped to the road network was recorded [26]. Agency-specific turnout times were then added, yielding the response time of each agency: travel time plus 2 min for EMS, plus 5 min for volunteer fire brigades and plus 0 min for police units. In the original analysis, only the ten stations closest by straight-line distance had been routed, in the direction from the incident to the station; the effect of both choices is reported in Table S7a.

The response time of a dispatch configuration was defined as the earliest arrival of any unit belonging to that configuration. Four configurations were evaluated at each of the simulated locations: EMS alone (group 1); EMS plus police, i.e., the minimum of the EMS and the police response time (group 2); EMS plus fire brigade, i.e., the minimum of the EMS and the fire brigade response time (group 3); and triple dispatch, i.e., the minimum of groups 2 and 3 (group 4). Response time in this model therefore denotes the interval from dispatch to arrival of the first unit at the incident coordinate. It does not include call-taking and dispatch processing, the time required to access the patient after arrival at the address, or the interval until the first chest compression is delivered.

### 2.8. Coverage Analysis

To describe geographical coverage independently of the sampled incident locations, drive-time isochrones were retrieved from ORS for every station and clipped to the state boundary. The isochrone ranges were chosen such that the total response time of each agency equaled 10 min once its turnout time was accounted for: 8 min of driving for EMS, 10 min for police units and 5 min for fire brigades. The union of the relevant surfaces represents the area of the state that can be reached within 10 min under each dispatch policy. For every surface the enclosed resident population was computed by overlaying the GHS-POP grid, so that coverage could be expressed both as a share of the area of the state and as a share of its inhabitants. Because isochrones and matrix travel times are derived from the same routing engine, they are not independent. The difference between the two coverage estimates was decomposed stepwise into contributions of station pre-selection, routing direction, threshold definition, raster-cell allocation and a remainder consistent with differences in isochrone geometry, the latter examined by intersecting the simulated locations themselves with the isochrones (Table S7a,b). Isochrones were recomputed for each turnout scenario of the sensitivity analysis. In both approaches, a location was counted as covered if its response time was ≤10 min.

### 2.9. Statistical Analysis

Response times are reported as median with interquartile range (IQR) and as mean, because the distributions were markedly right-skewed. As all four dispatch configurations were evaluated at every simulated location, the data are fully paired. Each co-dispatch configuration is defined as the earliest arrival among its units and always contains the ambulance service, so its response time cannot by construction exceed that of EMS alone; these comparisons were therefore not tested but described by the paired time gain (median, IQR and proportion of locations with a gain) and by the coverage gain in percentage points. The incremental value of triple dispatch was described in the same way against each dual configuration. The 95% confidence intervals (CIs) were obtained by paired bootstrap resampling of locations (2000 resamples, percentile method). Inferential testing was restricted to the comparison between the two dual configurations, in which either agency may prevail: because the paired differences were asymmetric and included exact ties, the sign test (ties excluded and reported) was used as the primary test, the Wilcoxon signed-rank test with three tie-handling rules (exclusion, Pratt, zero-splitting) as a sensitivity analysis, and the exact McNemar test for the difference in 10 min coverage; the matched-pairs rank-biserial correlation is reported as effect size. Uncertainty was reported separately by source. Sampling uncertainty was assessed with bootstrap CI and the 20 independent replicate samples. Turnout times were varied between 0 and 12 min for the fire brigade (0.5 min steps), 0 and 5 min for police and 1 and 2 min for EMS; for each value, coverage, paired time gains and the proportion of locations still benefiting were computed. Operational availability was examined in Monte Carlo simulations (500 replications) in which the availability of every station was drawn independently for each simulated location with a fixed probability (three levels: EMS 90%, 80% and 70%; police 85%, 70% and 50%; fire brigade 95%, 90% and 80%) and each agency responded with the fastest of its available stations; queueing, correlated unavailability of neighbouring units and repositioning were not modelled; in a night scenario without the daytime-only EMS stations, with stochastic turnout times (EMS uniform 1–3 min, police uniform 0–3 min, fire brigade log-normal with median 5 and 90th percentile 9 min), and with travel times multiplied by 0.85 (emergency driving) or 1.2 (winter conditions, congestion). For comparison with the regional registry, the proportion of locations reached by EMS alone within 8 min was computed (95% CI by the Wilson method). Results were stratified by political district, degree of urbanisation and whether EMS alone reached the location within 10 min. Locations for which no route could be computed were excluded from all analyses. Analyses were performed in Python 3 using pandas, NumPy 1.26.4 and SciPy 1.13.0; geoprocessing used GeoPandas 1.1.1 and rasterio 1.4.3, and maps were produced with matplotlib 3.8.4 and contextily 1.7.0. The complete code is provided as Code S1.

## 3. Results

Out of 500 simulated missions 499 could be analysed. One location in the Montafon region was excluded because no navigable road access existed for any of the three agencies; this location would only have been reachable by helicopter or on foot.

**Figure 2:**
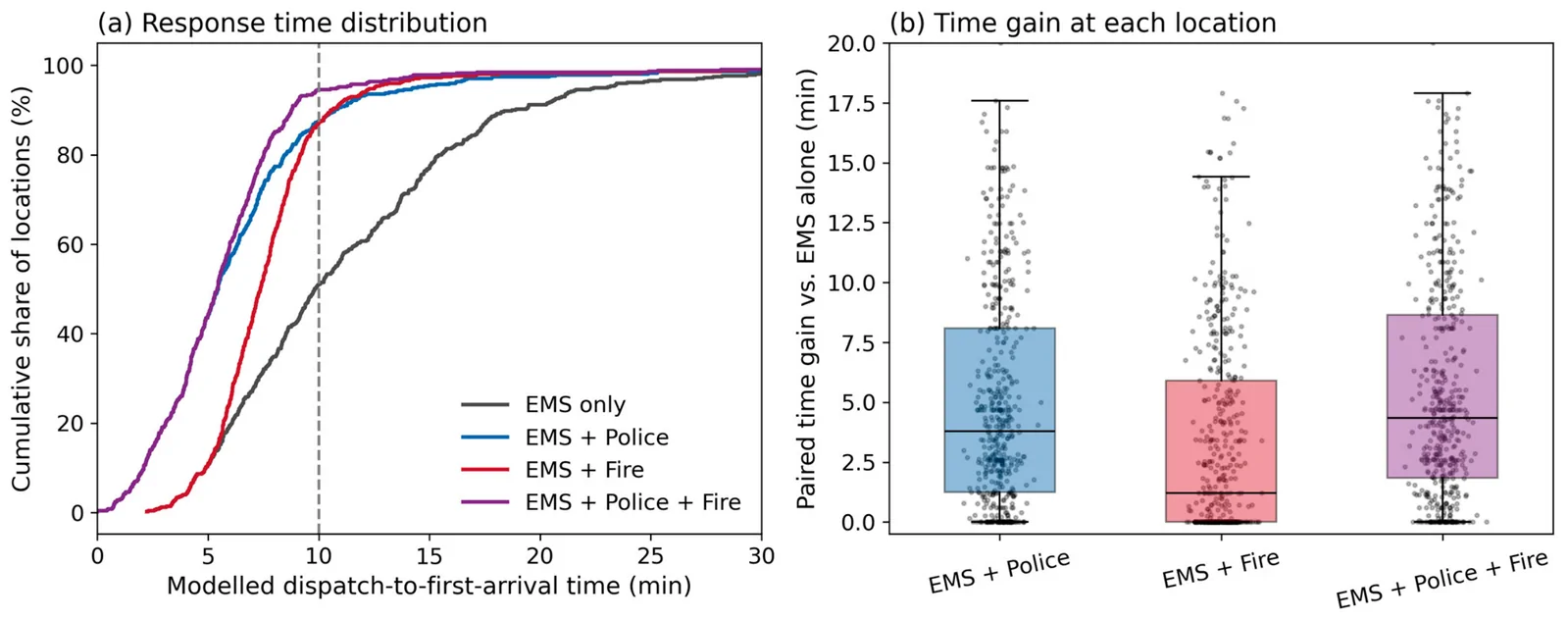
Response times by dispatch configuration across 499 simulated out-of-hospital cardiac arrest locations. (**a**) Cumulative distribution of the modelled dispatch-to-first-arrival time; the dashed line marks the 10 min threshold. (**b**) Paired time gain of each co-dispatch configuration over EMS alone at every location; boxes show median and interquartile range, whiskers extend to 1.5 times the interquartile range, and points show individual locations. No significance tests are shown, because co-dispatch cannot be slower than EMS alone by construction.

Median response time was 9.81 min (IQR 6.60–14.62; mean 11.91 min) when EMS were dispatched alone. Adding police reduced the median to 5.45 min (IQR 3.61–7.74; mean 6.85 min), adding the fire brigade to 7.36 min (IQR 6.00–8.77; mean 8.38 min), and triple dispatch produced the shortest median response time of 5.41 min (IQR 3.61–7.12; mean 6.26 min) (Table 1, Figure 2). Compared with EMS alone, the paired median time gain was 3.79 min (95% CI 3.26–4.29) for EMS plus police, 1.21 min (0.68–1.83) for EMS plus fire brigade and 4.34 min (3.80–4.67) for triple dispatch; a gain occurred at 86.6%, 57.7% and 89.2% of locations, respectively. In the only comparison in which either agency could prevail, EMS plus police was faster than EMS plus fire brigade at 69.1% of locations, slower at 20.0% and equal at 10.8% (sign test *p* < 0.001; median paired difference 1.59 min in favour of police, 95% CI 1.14–2.06; matched-pairs rank-biserial correlation r = −0.61); the Wilcoxon signed-rank test gave *p* < 0.001 under all three tie-handling rules.

With EMS alone, 255 of 499 locations (51.1%) were reached within 10 min and 174 (34.9%, 95% CI 30.8–39.2) within 8 min. Coverage rose to 436 locations (87.4%, 95% CI 84.4–90.0) with EMS plus police, 434 locations (87.0%, 84.0–90.0) with EMS plus fire brigade and 472 locations (94.6%, 92.6–96.6) with triple dispatch (Table 1). The two dual configurations thus had similar point estimates, with uncertainty compatible with differences in either direction (EMS plus police minus EMS plus fire brigade: 0.4 percentage points, 95% CI −2.8 to 3.8; exact McNemar test *p* = 0.91). Compared with EMS plus police, triple dispatch was faster at 20.0% of locations, with a median gain of 1.79 min at those locations, and reached 36 additional locations within 10 min (+7.2 percentage points, 95% CI 5.0–9.6); compared with EMS plus fire brigade, it was faster at 69.1% of locations (median gain 2.73 min) and reached 38 additional locations (+7.6 percentage points, 95% CI 5.4–10.0). The median response times of EMS plus police and triple dispatch therefore differed little, whereas their coverage differed by 7.2 percentage points: the fire brigade arrived before both EMS and police at one in five locations, mostly remote from a police station, where it often decided whether the threshold was met. In five locations (1.0%), even triple dispatch did not achieve arrival within 30 min: three at the Silvretta reservoir (Bielerhöhe) in the inner Montafon and two in the high-alpine area north of the Klostertal near the Formarinsee, which the routing engine reached only via Lech.

**Figure 3:**
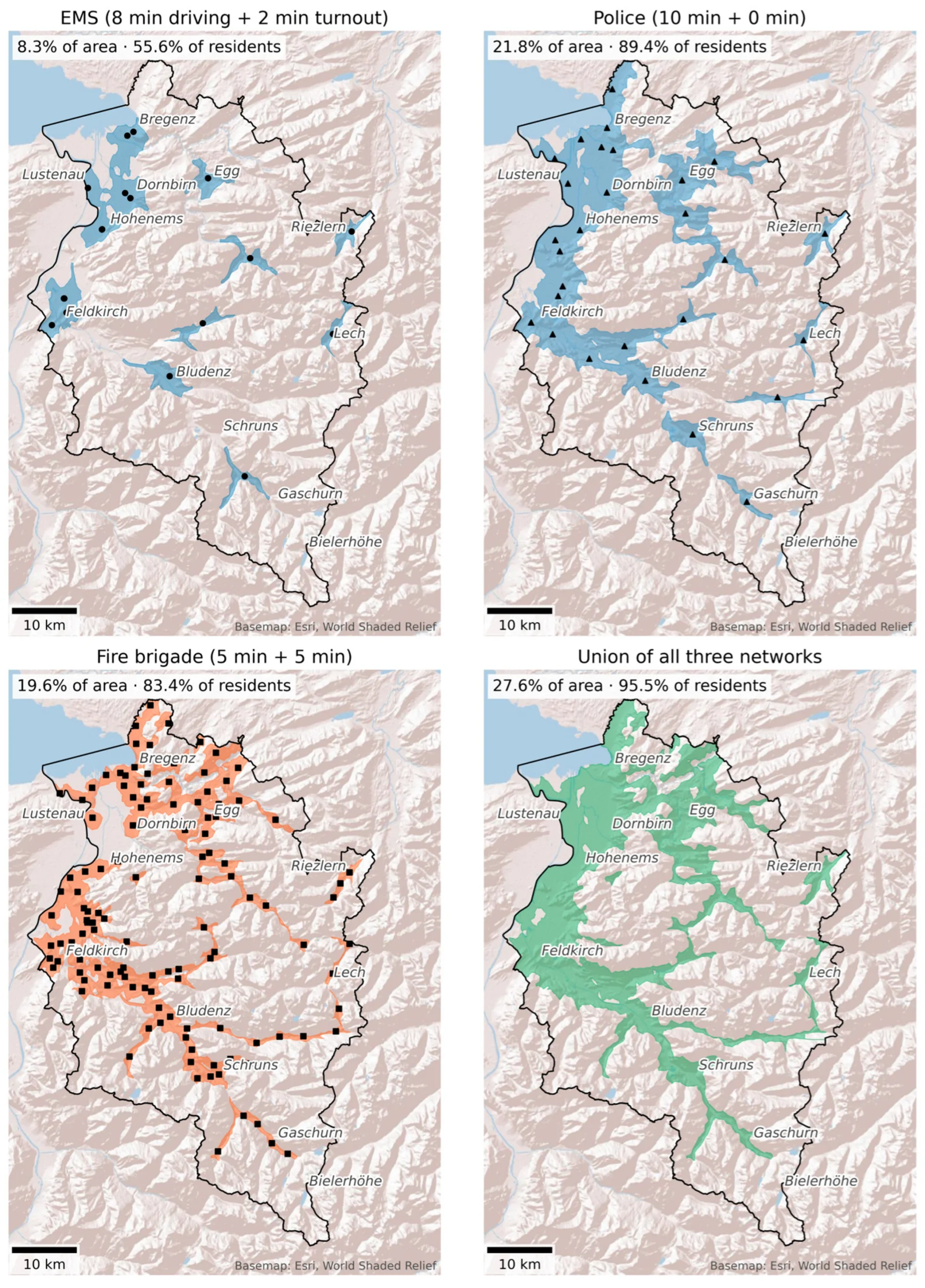
Area of Vorarlberg reachable within 10 min by each responder network when dispatched alone, derived from drive-time isochrones clipped to the state boundary. Isochrone ranges account for agency-specific turnout times (EMS 8 min of driving plus 2 min turnout; police 10 min plus 0 min; fire brigade 5 min plus 5 min). The fourth panel shows the union of all three surfaces, which corresponds to the triple-dispatch configuration. Isochrones were recomputed with the corrected station list. Background: Esri, World Shaded Relief; place names added by the authors.

Considered individually, police units arrived before EMS in 432 of 499 missions (86.6%) and fire brigades in 288 (57.7%); when both agencies were dispatched together with EMS, a non-EMS unit was first on scene in 445 missions (89.2%). The isochrone analysis showed the same pattern geographically: the 10 min surface of the police network covered the valley floors and the main transport axes, whereas the denser fire station network extended coverage into side valleys that neither EMS nor police reached within 10 min (Figure 3). Of the area of the state, 217 km^2^ (8.3%) lay within 10 min of an ambulance station, 569 km^2^ (21.8%) within 10 min of a police station and 510 km^2^ (19.6%) within 10 min of a fire station. Combining EMS with police covered 587 km^2^ (22.6%), EMS with the fire brigade 586 km^2^ (22.5%), and all three agencies together 717 km^2^ (27.6%) of the state (Table 2).

Surfaces are drive-time isochrones clipped to the state boundary; populations were computed by overlaying the 100 m GHS-POP grid. The police-only and fire-brigade-only rows describe the geographical reach of those networks and do not correspond to dispatch configurations that were evaluated, since the ambulance service is dispatched to every out-of-hospital cardiac arrest. Isochrones were recomputed with the corrected station list; residential population from GHS-POP, epoch 2030.

Weighted by resident population rather than by area, the same surfaces covered 55.6% of the population for EMS alone, 90.9% with police added, 91.9% with the fire brigade added and 95.5% under triple dispatch, leaving approximately 17,800 residents outside the 10 min surface of any configuration. The police network covered 12% more of the area of the state than the fire brigade network (21.8% versus 19.6%) and a larger share of its residents (89.4% versus 83.4%), reflecting police stations along the main transport axes and fire stations within settlements. The isochrone estimates exceeded the corresponding mission-based estimates by 0.9 to 4.9 percentage points. In a stepwise decomposition (Table S7a), station pre-selection, routing direction and threshold definition together accounted for at most 1.2 percentage points and sampling variation for 0.3 to 1.5 percentage points. When the simulated locations themselves were intersected with the isochrones (Table S7a), 3 to 22 locations per configuration lay inside an isochrone although their matrix response time exceeded 10 min; these locations were close to the threshold (median 10.4–11.3 min) and close to a road (median snapping distance 11–22 m). This pattern is consistent with differences in isochrone geometry, the polygons including areas just beyond the threshold, although the mechanism of each individual discrepancy cannot be identified; the isochrone estimates should therefore be read as the more generous geographical summary, and the matrix-based estimates remain the primary measure.

The benefit attributed to the fire brigade depended strongly on the assumed turnout time, whereas the benefit of triple dispatch was more robust (Table S1, Figure S1). Reducing the fire brigade turnout time from 5 min to 3 min raised the 10 min coverage of EMS plus fire brigade from 87.0% to 93.4%, and increasing it to 8 min lowered coverage to 62.7%, still 11.6 percentage points above EMS alone; fire brigade co-dispatch added coverage up to a turnout time of about 9.5 min. Beyond this point the fire brigade no longer added locations within 10 min but still arrived first at 28.7% of locations at a turnout time of 10 min (median gain there 3.84 min) and at 21.2% at 12 min (2.83 min). The corresponding coverage under triple dispatch was 95.2%, 94.6% and 90.2% at fire brigade turnout times of 3, 5 and 8 min. Increasing the police turnout time from 0 to 2 min reduced coverage of EMS plus police from 87.4% to 79.6% and that of triple dispatch to 92.2%. Under the least favourable combination examined, with EMS at 1 min, the fire brigade at 8 min and police at 2 min, triple dispatch reached 85.2% of locations within 10 min against 56.5% for EMS alone. Recomputed isochrones showed the same dependence (Table S2b).

Sampling uncertainty was small relative to these effects (Table S4). Across 20 independent samples, 10 min coverage varied with a standard deviation of 1.2 to 2.4 percentage points (mean 49.9% for EMS alone and 94.7% for triple dispatch), and samples drawn from the 2020 and 2025 population grids gave a coverage of 53.4% and 50.5% for EMS alone and 95.2% and 94.0% for triple dispatch. Restricting the routing to the ten stations closest by straight-line distance, as in the original analysis, would have missed the fastest station at 0.2% of locations for EMS, at none for police and at 0.8% for the fire brigade, overestimating the response time at these locations by up to 2.2 and 21.1 min, respectively.

Operational availability changed the relative contribution of the two agencies (Table S5). With unit availability of 80% for EMS, 70% for police and 90% for fire brigades, 10 min coverage was 44.7% for EMS alone, 77.8% for EMS plus police, 82.7% for EMS plus fire brigade and 91.2% for triple dispatch; with 50% police availability, the corresponding values were 40.8%, 68.8%, 78.3% and 87.0%. Closing the daytime-only EMS stations at night reduced EMS-alone coverage from 51.1% to 48.7% without affecting triple dispatch (94.6%). Stochastic turnout times lowered coverage by up to 5.5 percentage points (EMS plus police 81.9%), and travel times multiplied by 0.85 or 1.2 yielded 95.6% and 90.4% for triple dispatch.

The benefit differed markedly between areas (Table S6). In rural areas, EMS alone reached 16.5% of locations within 10 min, EMS plus police 55.6%, EMS plus fire brigade 70.7% and triple dispatch 79.7%, with a median time gain of 6.6 min for triple dispatch. In urban clusters, EMS alone already reached 63.7% of locations, and police co-dispatch raised coverage to 98.9% (fire brigade 92.9%, triple dispatch 100%). By district, the coverage gain of triple dispatch ranged from 9.0 percentage points in Dornbirn, where EMS alone reached 89.2% of locations, to 52.2 and 61.5 percentage points in Bludenz and Feldkirch.

## 4. Discussion

This simulation study shows that co-dispatching police units and volunteer fire brigades together with EMS could markedly shorten modelled response times to OHCA in Vorarlberg, a state of 2601 km^2^ with approximately 412,000 inhabitants distributed across a mixed urban and alpine landscape [18,19]. The effect was present for both additional agencies and was most pronounced when both were dispatched together with EMS, with a median time gain of 4.34 min (95% CI 3.80–4.67) compared with EMS alone. To our knowledge, this is the first study to evaluate both police and fire services as a first responder system across an entire Austrian federal state.

The two agencies contributed in different ways, and distinguishing the mechanisms is central to interpreting these results. Police units delivered the larger median time gain and arrived before EMS in 86.6% of missions. In the model, this advantage follows from three assumptions rather than from a demonstrated effect of field deployment: police stations lie along the main transport axes, police units were assigned no turnout time, and they were always available; patrol movements were not modelled. When police availability was reduced to 70% or 50%, police co-dispatch reached 77.8% or 68.8% of locations within 10 min, fewer than fire brigade co-dispatch (82.7% and 78.3%). Fire brigades produced a smaller median gain, since volunteer members must first travel to the station, but their network is more than four times denser (121 versus 28 stations) and reaches side valleys that are remote from both ambulance and police stations; in rural areas, fire brigade co-dispatch reached 70.7% of locations within 10 min, compared with 55.6% for police. Across the state, the two dual configurations had similar point estimates for 10 min coverage (87.4% and 87.0%), with uncertainty compatible with differences in either direction. Speed and coverage are thus distinct properties of the two networks, which are complementary rather than interchangeable: only their combination reached 94.6% of locations within 10 min.

Police cars in Vorarlberg already carry AEDs and officers receive mandatory basic life support and tactical medicine training, which makes law enforcement a well-prepared first responder resource. Police co-dispatch is an established practice in many regions worldwide; within Austria it is systematically implemented only in Vienna, where both fire services and police are dispatched to every cardiac arrest. Several studies have reported higher survival with police co-dispatch and with police-based AED programmes [27,28].

In contrast, most Austrian firefighters are volunteers with civil occupations. They are not immediately available but must first travel to the fire station, put on protective equipment and only then proceed to the scene. Compared location by location, the police therefore arrived a median of 1.59 min earlier than the fire brigade. This difference is small in absolute terms but is relevant in the situations in which fire units are first on scene, which in our model was the case in more than half of all missions. A nationwide Swedish study of dual dispatch demonstrated a median gain of approximately one minute in time to arrival together with a survival benefit [28], suggesting that time gains of this magnitude can be relevant; clinical outcomes, however, were not assessed in the present study.

An important feature of our study is that the simulated OHCA locations span both urban and rural areas, in which population density and the spacing of EMS, fire and police stations differ substantially. Rural areas are characterised by lower exposure of bystanders to OHCA, longer response times and consequently lower survival. With 121 stations, the fire brigades provide the highest station density across the state and reach even small side valleys. The stratified results support this: in rural areas, triple dispatch raised 10 min coverage from 16.5% to 79.7% and shortened the modelled response time by a median of 6.6 min, whereas in the district of Dornbirn the coverage gain was 9.0 percentage points. Implementing firefighters as first responders could therefore be particularly valuable in those areas in which the current EMS response is slowest, with the potential to shorten the interval to first responder arrival where the gain is largest. The isochrone data show how little of that peripheral reach the current system already possesses: adding the ambulance stations to the police network enlarges its 10 min surface only from 21.8% to 22.6% of the state, so the benefit of co-dispatch accrues almost entirely outside the areas that EMS already serves well. Conversely, the five locations that remained unreachable within 30 min even under triple dispatch illustrate that no ground-based dispatch strategy can fully compensate for extreme alpine remoteness.

Beyond arrival time, the number of trained responders on scene affects the quality of resuscitation. Several studies indicate that a team of at least four to five providers is associated with a higher chest compression fraction (CCF), reduced rescuer fatigue during prolonged resuscitation and faster delivery of advanced life support interventions, although findings on crew size are not uniform [29,30,31]. Dispatching fire brigade or police personnel could therefore, as a hypothesis for future studies, also improve these process metrics by increasing the number of hands available at the scene; time to CPR initiation, time to first shock, compression quality and survival with favourable neurological outcome require direct measurement in implementation studies.

Implementing additional first responders is a necessary component of an effective system saving lives, but its efficiency depends on several preconditions. First, the dispatch centre must reliably recognise OHCA; robust quality assurance metrics and standardised training for telecommunicators are required to increase T-CPR delivery rates, which in Vorarlberg currently stand at 24.1% of all treated cardiac arrests (72 of 299). Alerting of all responder groups must occur without delay [26]. Second, the deployment of certified lay responders and the strategic mobilisation of AEDs through smartphone-based alerting applications represent an existing regional framework that warrants expansion. Third, the psychological burden on non-medical responders must be addressed: in an interview study by Hasselqvist-Ax et al., firefighters and police officers felt well prepared for OHCA missions and rated their self-efficacy as high, provided that CPR training was adequate and the information given by the dispatch centre was complete [32].

A recent analysis of OHCA epidemiology in Vienna showed stagnating overall survival despite an ageing population, while survival in the subgroup with an initially shockable rhythm improved compared with 2009–2013, a period during which fire brigade and police first responder programmes and an active extracorporeal CPR (ECPR) programme were introduced [33,34]. In Vorarlberg, the regional hospital provides ECPR under strict inclusion criteria that require a witnessed arrest and a minimal no-flow interval. Achieving outcomes comparable to Vienna therefore requires optimisation of every prehospital interval. As one of our 500 simulated locations was not reachable by road at all, HEMS remains a relevant complementary resource; however, median HEMS response times to OHCA in Austria are 9–12 min because of start and landing phases [35], which is too long to serve as the primary bridging strategy. HEMS is critical for transporting selected OHCA patients to ECPR centres, but only if it is co-dispatched with a ground crew, and ECPR remains feasible only when first responders and bystanders have kept the no-flow interval short.

### Limitations

The model is a deliberately static representation of a system that is in constant motion. Every unit was routed from its station and, in the main analysis, treated as unconditionally available, whereas police patrols and ambulances are frequently already on the road, committed to another assignment, or returning from one; the availability scenarios show that this assumption favours police co-dispatch in particular. Routing police from their station is not necessarily conservative, because a patrol car may be closer to or farther from an incident than its station; together with the assumption of no turnout time, the direction of the resulting error for police is unknown. Driving times came from a routing engine parameterised for ordinary passenger cars, so neither the time gained through warning devices and emergency driving privileges nor the time lost to congestion, winter road conditions or closures enter the estimate. In the model, EMS reached 34.9% of locations within 8 min, whereas in the regional registry the first vehicle, including existing first responders, arrived within 8 min of the alarm in 73.2% of resuscitations in 2024. Because the two figures differ in the responding units and in the incident locations, this comparison cannot identify the direction or magnitude of the error in the modelled EMS travel times, and the calibration of EMS travel times therefore remains uncertain. Three of the five locations not reached within 30 min lie at the Silvretta reservoir; the routing engine apparently treated the Silvretta High Alpine Road from the Montafon as impassable and routed via Tyrol. As this road is closed in winter, these times reflect a seasonal access restriction rather than year-round conditions.

Among these assumptions the turnout time of the volunteer fire brigades carries the most weight, since it enters the fire brigade result almost linearly and rests on operational experience and a single published source. The sensitivity analysis makes that dependency explicit: at three minutes the fire brigade configuration reaches 93.4% of locations within ten minutes, at the assumed five minutes 87.0%, and at eight minutes 62.7%; it adds coverage over EMS alone up to a turnout time of about 9.5 min and still arrives first at some locations beyond that. The corresponding values for triple dispatch are 95.2%, 94.6% and 90.2%, so while the case for the fire brigade alone is conditional on a short turnout, the case for combined dispatch is not. The isochrone surfaces placed population coverage up to five percentage points above the mission-based estimates; this difference is consistent with differences in isochrone geometry, but the analysis cannot identify the mechanism of each discrepancy or exclude a contribution of road snapping; the matrix-based estimates therefore remain the primary measure, and the isochrone figures should be read as the more generous geographical summary. A turnout time of zero minutes was assumed for the police, as patrol cars are typically already on the road at the time of the alert. Assuming that patrol cars are evenly distributed around their police station, the station’s location was used as a proxy for their mean position; at an individual incident, a patrol car may be closer to or farther from the incident than its station.

What the simulated locations represent limits the reach of the findings in a different way. They were drawn from a modelled residential population grid (epoch 2030, chosen for the planning horizon; results with the 2020 and 2025 epochs were similar), which assumes that cardiac arrest risk follows where people are registered rather than where they are, and takes no account of age structure, workplaces, or the substantial tourist and transit population of the alpine valleys, the very areas in which the co-dispatch benefit proved largest. Tourism multiplies the population present in these valleys during the winter and summer seasons, which suggests that residential weighting underestimates the share of arrests in peripheral areas, whereas daytime workplace populations shift risk towards the Rhine Valley; age-specific risk was not modelled. Reporting coverage by area alongside coverage by population is a partial remedy but no substitute for exposure data. The endpoint is likewise narrower than survival, ending at arrival at the incident coordinate and excluding call-taking, dispatch processing, the time needed to locate and reach the patient within a building, and the interval until the first compression or first shock. It also treats arriving units as interchangeable when they are not, since a police officer with an automated external defibrillator and basic life support training and an ambulance crew with advanced life support capability differ in what they can do once on scene. The results therefore describe the potential to shorten the time to first responder arrival, and possibly the no-flow interval, rather than an effect on outcome.

Neither app-based alerts for lay rescuers or off-duty firefighters nor the strategy of bypassing the fire stations and immediate travel to the emergency location were included into this simulation. These considerations could serve for additional studies to further reduce the travel times. First responder schemes, which already exist in Vorarlberg, were not modelled either; the incremental benefit of police and fire brigade co-dispatch relative to the existing responder system was therefore not estimated.

Nothing in this analysis addresses what a co-dispatch policy would cost. The opportunity cost of committing police and fire resources to medical missions, the consequences of dispatching them to suspected arrests that turn out not to be one, the training and equipment burden, and the psychological load on volunteers who did not choose a medical career all bear on whether such a programme is sustainable, and each needs examination before implementation.

## 5. Conclusions

Under fixed station locations, continuous unit availability and assumed agency-specific turnout times, police and fire brigade co-dispatch increased modelled ten-minute coverage compared with EMS alone. Operational validation is required before these estimates can inform regional implementation. The estimates refer to the station inventory at the time of the analysis and would need to be updated if stations are added, closed or relocated.

In this model, triple dispatch raised the proportion of locations reached within 10 min from 51.1% to 94.6% and shortened the modelled response time by a median of 4.3 min. Police co-dispatch produced the larger median time gain, a result that depends on the assumed immediate availability of police units, whereas the denser fire station network contributed most in rural areas; the coverage point estimates of the two dual configurations were similar, which does not demonstrate equivalence, and their combination reached the most locations. The benefit of fire brigade co-dispatch decreased steadily with longer turnout times but added coverage up to a turnout time of about 9.5 min.

These findings suggest that integrating multi-agency first responder tiers into standard dispatch protocols could shorten the interval preceding the arrival of advanced life support. Prospective, real-world implementation studies are warranted to validate these modelled time gains, to measure time to CPR initiation and first shock, compression quality and neurologically intact survival to discharge, and to define the operational integration of volunteer networks into the regional emergency response system.

## Figures and Tables

**Table 1:** Response times and 10 min coverage of the four dispatch configurations across 499 simulated out-of-hospital cardiac arrest locations in Vorarlberg, Austria.

Dispatch Configuration	Median (IQR), Min	Mean (Min)	Median Gain vs. EMS Alone (IQR) [95% CI], min	Reached ≤10 min, *n* (%) [95% CI]	First on Scene Before EMS, *n* (%)	Coverage Gain vs. EMS Alone, pp (95% CI)
EMS only	9.81 (6.60–14.62)	11.91	reference	255 (51.1) [46.7–55.3]	not applicable	reference
EMS + police	5.45 (3.61–7.74)	6.85	3.79 (1.25–8.09) [3.26–4.29]	436 (87.4) [84.4–90.0]	432 (86.6)	+36.3 (32.3–40.3)
EMS + fire brigade	7.36 (6.00–8.77)	8.38	1.21 (0.00–5.91) [0.68–1.83]	434 (87.0) [84.0–90.0]	288 (57.7)	+35.9 (31.9–40.1)
EMS + police + fire brigade	5.41 (3.61–7.12)	6.26	4.34 (1.85–8.66) [3.80–4.67]	472 (94.6) [92.6–96.6]	445 (89.2)	+43.5 (39.3–47.9)

EMS, emergency medical services; CI, confidence interval; pp, percentage points. Response time is the interval from dispatch to arrival of the first unit of the respective configuration. Gains are paired differences from EMS alone; 95% CI from 2000 paired bootstrap resamples. No significance tests are reported for comparisons with EMS alone, because co-dispatch cannot be slower than EMS alone by construction.

**Table 2:** Area and resident population of Vorarlberg reachable within 10 min, by responder network and dispatch configuration.

Surface	Area (km^2^)	Area (% of State)	Population	Population (%)
EMS only	216.6	8.3	219,595	55.6
Police only	568.6	21.8	352,997	89.4
Fire brigade only	509.9	19.6	329,206	83.4
EMS + police	587.4	22.6	358,966	90.9
EMS + fire brigade	586.5	22.5	362,737	91.9
EMS + police + fire brigade	717.2	27.6	376,977	95.5

## Data Availability

The 500 simulated incident locations, the station inventory with coordinates (Table S3), the station-to-location travel-time matrices and the complete analysis code (Code S1) are provided as Supplementary Materials (Data S1), so that all reported results can be reproduced without new routing requests. Routing used the public OpenRouteService API (Heidelberg Institute for Geoinformation Technology) in October 2026. Further inquiries can be directed to the corresponding authors.

## Supplementary Materials

The following supporting information can be downloaded at: https://www.mdpi.com/article/10.3390/jcm15218237/s1, Table S1: Sensitivity of coverage and paired time gains to the assumed turnout times; Figure S1: Coverage and paired time gains as a function of the assumed fire brigade turnout time; Table S2: (a) Area and resident population covered by every isochrone surface, and (b) isochrone coverage for each turnout scenario; Table S3: Station inventory; Table S4: Monte Carlo uncertainty: (a) bootstrap confidence intervals, (b) replicate samples, and (c) population epochs; Table S5: Unit availability, stochastic turnout and travel-time scenarios; Table S6: Results by district, degree of urbanisation and EMS response stratum; Table S7: (a) Decomposition of the difference between isochrone- and mission-based coverage, and (b) same-point comparison of isochrones and matrix travel times; Table S8: Comparison of the dual configurations; Table S9: Incremental value of triple dispatch over each dual configuration; Code S1: Analysis code; Data S1: Simulated incident locations, station coordinates and station-to-location travel-time matrices.

## Abbreviations

The following abbreviations are used in this manuscript:

OHCA	Out-of-Hospital Cardiac Arrest
EMS	Emergency Medical Services
CPR	Cardiopulmonary Resuscitation
ILCOR	International Liaison Committee on Resuscitation
ERC	European Resuscitation Council
ROSC	Return of Spontaneous Circulation
RFL	Rettungs- und Feuerwehrleitstelle (emergency medical and fire dispatch centre)
NEF	Notarzteinsatzfahrzeug (physician-staffed emergency response car)
AED	Automated External Defibrillator
BLS	Basic Life Support
CCF	Chest Compression Fraction
T-CPR	Telephone-Assisted Cardiopulmonary Resuscitation
ECPR	Extracorporeal Cardiopulmonary Resuscitation
GHS-POP	Global Human Settlement Population Grid
HEMS	Helicopter Emergency Medical Services
IQR	Interquartile Range
ORS	OpenRouteService
