# Supplementary material for: Modelled Response Times for Police and Fire Brigade Co-Dispatch to Out-of-Hospital Cardiac Arrest: A Geospatial Simulation Study in Vorarlberg, Austria
Source: J Clin Med. 2026 Oct 22;15(21):8237. doi: 10.3390/jcm15218237 (PMC13764481; doi:10.3390/jcm15218237)
Supplement: Supplementary file 1 [file jcm-15-08237-s001.zip › Supplementary_Materials_R2.pdf]

Supplementary Materials

Modelled Response Times for Police and Fire Brigade Co-Dispatch to Out-of-Hospital Cardiac Arrest: A Geospatial Simulation Study in Vorarlberg, Austria

Table S1. Sensitivity of coverage and paired time gains to the assumed turnout times. EMS turnout 2 min; coverage threshold ≤10 min. 'Fire first' is the share of locations at which the fire brigade arrives before EMS. The complete results (fire brigade 0–12 min in 0.5-min steps, police 0–5 min, EMS 1 and 2 min) are reproduced by Code S1 from Data S1.

Police turnout (min)	Fire turnout (min)	EMS only ≤10 min (%)	EMS + fire ≤10 min (%)	Coverage gain (pp)	Median gain (min)	Fire first (%)	Median gain if fire first (min)	EMS + police ≤10 min (%)	Triple ≤10 min (%)	Triple coverage gain (pp)
0.00	0.00	51.10	96.59	45.49	6.21	93.39	6.76	87.37	96.99	45.89
0.00	1.00	51.10	95.59	44.49	5.21	89.18	6.13	87.37	96.39	45.29
0.00	2.00	51.10	94.79	43.69	4.21	80.96	5.66	87.37	95.79	44.69
0.00	3.00	51.10	93.39	42.28	3.21	71.34	5.45	87.37	95.19	44.09
0.00	4.00	51.10	91.38	40.28	2.21	63.53	5.24	87.37	94.99	43.89
0.00	5.00	51.10	86.97	35.87	1.21	57.72	5.00	87.37	94.59	43.49
0.00	6.00	51.10	80.36	29.26	0.21	51.50	4.53	87.37	93.59	42.48
0.00	7.00	51.10	72.95	21.84	0.00	44.89	4.61	87.37	91.38	40.28
0.00	8.00	51.10	62.73	11.62	0.00	39.48	4.40	87.37	90.18	39.08
0.00	9.00	51.10	55.31	4.21	0.00	33.87	4.10	87.37	88.38	37.27
0.00	10.00	51.10	51.10	0.00	0.00	28.66	3.84	87.37	87.37	36.27
0.00	11.00	51.10	51.10	0.00	0.00	24.65	3.29	87.37	87.37	36.27
0.00	12.00	51.10	51.10	0.00	0.00	21.24	2.83	87.37	87.37	36.27
2.00	0.00	51.10	96.59	45.49	6.21	93.39	6.76	79.56	96.59	45.49
2.00	1.00	51.10	95.59	44.49	5.21	89.18	6.13	79.56	95.79	44.69
2.00	2.00	51.10	94.79	43.69	4.21	80.96	5.66	79.56	94.99	43.89
2.00	3.00	51.10	93.39	42.28	3.21	71.34	5.45	79.56	93.99	42.89
2.00	4.00	51.10	91.38	40.28	2.21	63.53	5.24	79.56	93.39	42.28
2.00	5.00	51.10	86.97	35.87	1.21	57.72	5.00	79.56	92.18	41.08
2.00	6.00	51.10	80.36	29.26	0.21	51.50	4.53	79.56	89.98	38.88
2.00	7.00	51.10	72.95	21.84	0.00	44.89	4.61	79.56	86.57	35.47
2.00	8.00	51.10	62.73	11.62	0.00	39.48	4.40	79.56	83.37	32.26
2.00	9.00	51.10	55.31	4.21	0.00	33.87	4.10	79.56	80.96	29.86
2.00	10.00	51.10	51.10	0.00	0.00	28.66	3.84	79.56	79.56	28.46
2.00	11.00	51.10	51.10	0.00	0.00	24.65	3.29	79.56	79.56	28.46
2.00	12.00	51.10	51.10	0.00	0.00	21.24	2.83	79.56	79.56	28.46

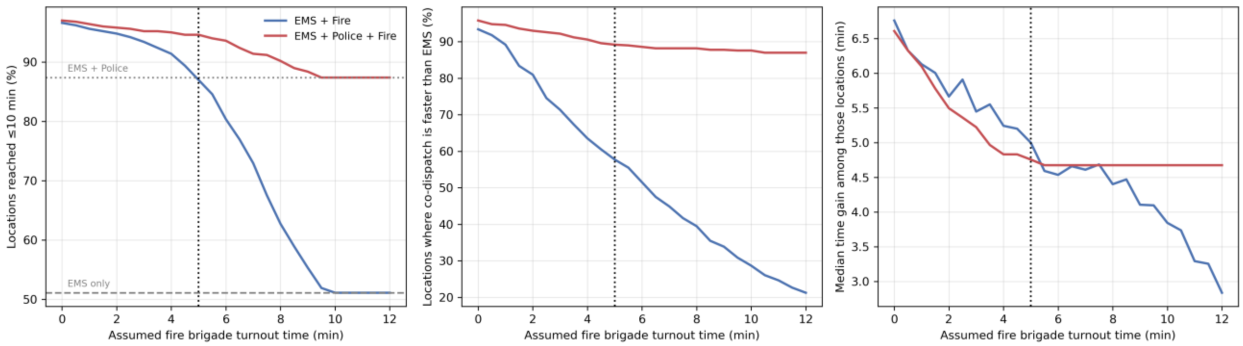

Figure S1. Coverage and paired time gains as a function of the assumed fire brigade turnout time. Left: 10-minute coverage; centre: share of locations at which co-dispatch arrives before EMS; right: median time gain at those locations. Dotted vertical line: base case (5 min).

Table S2a. Area and resident population covered by every isochrone surface. Isochrones with a driving time of 10 min minus turnout time, clipped to the state boundary; areas in ETRS89-LAEA (EPSG:3035), population from GHS-POP E2030. Last column: share of simulated locations reached ≤10 min in the matrix-based analysis.

Isochrone surface	Area (km^2)	Area (% of state)	Residents (n)	Residents (% of state)	Simulated locations reached ≤10 min, matrix analysis (%)
EMS	217	8.30	219,595	55.60	51.10
Police	569	21.80	352,997	89.40	–
Fire	510	19.60	329,206	83.40	–
EMS + Police	587	22.60	358,966	90.90	87.40
EMS + Fire	586	22.50	362,736	91.90	87.00
EMS + Police + Fire	717	27.60	376,977	95.50	94.60

Table S2b. Isochrone coverage for each turnout scenario. Share of residents (%) within the isochrones recomputed for each scenario.

Scenario	EMS turnout (min)	Police turnout (min)	Fire brigade turnout (min)	EMS alone (% of residents)	EMS + police (% of residents)	EMS + fire brigade (% of residents)	triple dispatch (% of residents)
Base	2	0	5	55.60	90.90	91.90	95.50
Fire 3 min	2	0	3	55.60	90.90	95.70	96.60
Fire 8 min	2	0	8	55.60	90.90	70.00	92.50
Police 2 min	2	2	5	55.60	85.40	91.90	94.10
EMS 1 min, fire 8, police 2	1	2	8	60.60	86.30	73.50	89.20

Table S3. Station inventory. Agency, name, vehicle type, coordinates (WGS 84), operating hours as modelled, capability to start CPR and to defibrillate, and source of coordinates.

Agency	Station	Vehicle type	Longitude (WGS 84)	Latitude (WGS 84)	Operating hours (as modelled)	CPR/AED capability	Coordinate source
EMS	Rotkreuz Ortsstelle Au	ambulance	9.98353	47.32344	24 h	CPR + defibrillation (ambulance: AED/monitor-defibrillator; NEF: ALS)	RFL Vorarlberg station list 2026
EMS	Rotkreuz-Abteilung Bludenz	ambulance	9.82001	47.16055	24 h	CPR + defibrillation (ambulance: AED/monitor-defibrillator; NEF: ALS)	RFL Vorarlberg station list 2026
EMS	Rotkreuz-Abteilung Bregenz	ambulance	9.73379	47.49204	24 h	CPR + defibrillation (ambulance: AED/monitor-defibrillator; NEF: ALS)	RFL Vorarlberg station list 2026
EMS	Rotkreuz-Abteilung Dornbirn	ambulance	9.72941	47.41309	24 h	CPR + defibrillation (ambulance: AED/monitor-defibrillator; NEF: ALS)	RFL Vorarlberg station list 2026
EMS	Rotkreuz-Abteilung Egg	ambulance	9.89786	47.43364	24 h	CPR + defibrillation (ambulance: AED/monitor-defibrillator; NEF: ALS)	RFL Vorarlberg station list 2026
EMS	Rotkreuz-Abteilung Feldkirch	ambulance	9.61001	47.24825	24 h	CPR + defibrillation (ambulance: AED/monitor-defibrillator; NEF: ALS)	RFL Vorarlberg station list 2026
EMS	Landesverband Vorarlberg	ambulance	9.60454	47.26787	daytime only (modelled 24 h)	CPR + defibrillation (ambulance: AED/monitor-defibrillator; NEF: ALS)	RFL Vorarlberg station list 2026
EMS	Rotkreuz Krankentransportzentrale	ambulance	9.60569	47.26799	daytime only (modelled 24 h)	CPR + defibrillation (ambulance: AED/monitor-defibrillator; NEF: ALS)	RFL Vorarlberg station list 2026
EMS	Rotkreuz-Abteilung Hohenems	ambulance	9.68219	47.36302	24 h	CPR + defibrillation (ambulance: AED/monitor-defibrillator; NEF: ALS)	RFL Vorarlberg station list 2026
EMS	Rotkreuz Ortsstelle Lech	ambulance	10.15219	47.21869	24 h	CPR + defibrillation (ambulance: AED/monitor-defibrillator; NEF: ALS)	RFL Vorarlberg station list 2026
EMS	Rotkreuz-Abteilung Lustenau	ambulance	9.65373	47.42014	24 h	CPR + defibrillation (ambulance: AED/monitor-defibrillator; NEF: ALS)	RFL Vorarlberg station list 2026
EMS	Aussenstelle Bludenz St. Gallenkirch	ambulance	9.97240	47.02205	24 h	CPR + defibrillation (ambulance: AED/monitor-defibrillator;	RFL Vorarlberg station list 2026

						NEF: ALS)	
EMS	Aussenstelle Bludenz Sonntag	ambulance	9.88744	47.23368	daytime only (modelled 24 h)	CPR + defibrillation (ambulance: AED/monitor-defibrillator; NEF: ALS)	RFL Vorarlberg station list 2026
EMS	NEF Bregenz	physician response car (NEF)	9.74608	47.49728	24 h	CPR + defibrillation (ambulance: AED/monitor-defibrillator; NEF: ALS)	RFL Vorarlberg (manual entry)
EMS	NEF Dornbirn	physician response car (NEF)	9.74004	47.40621	24 h	CPR + defibrillation (ambulance: AED/monitor-defibrillator; NEF: ALS)	RFL Vorarlberg (manual entry)
EMS	NEF Feldkirch	physician response car (NEF)	9.58015	47.23108	24 h	CPR + defibrillation (ambulance: AED/monitor-defibrillator; NEF: ALS)	RFL Vorarlberg (manual entry)
EMS	NEF Hohenems	physician response car (NEF)	9.68232	47.36326	24 h	CPR + defibrillation (ambulance: AED/monitor-defibrillator; NEF: ALS)	RFL Vorarlberg (manual entry)
EMS	KWT RD	ambulance	10.19023	47.36028	24 h	CPR + defibrillation (ambulance: AED/monitor-defibrillator; NEF: ALS)	RFL Vorarlberg (manual entry)
Police	PI Schruns Außenstelle Gaschurn	police patrol car (with AED)	10.02670	46.98680	24 h	CPR + AED (every patrol car carries an AED)	original list (Google Maps)
Police	PI Schruns	police patrol car (with AED)	9.91680	47.08010	24 h	CPR + AED (every patrol car carries an AED)	original list (Google Maps)
Police	PI Klösterle	police patrol car (with AED)	10.08915	47.13155	24 h	CPR + AED (every patrol car carries an AED)	Kloesterle 59b, 6754 Kloesterle (verified by authors, 13 Oct 2026)
Police	PI Bludenz	police patrol car (with AED)	9.82010	47.15440	24 h	CPR + AED (every patrol car carries an AED)	original list (Google Maps)
Police	PI Lech am Arlberg	police patrol car (with AED)	10.14220	47.21040	24 h	CPR + AED (every patrol car carries an AED)	original list (Google Maps)
Police	PI Thüringen	police patrol car (with AED)	9.77810	47.20230	24 h	CPR + AED (every patrol car carries an AED)	original list (Google Maps)
Police	PI Nenzing	police patrol car (with AED)	9.70628	47.18484	24 h	CPR + AED (every patrol car carries an AED)	original list (Google Maps)
Police	PI Frastanz	police patrol car (with AED)	9.63180	47.21850	24 h	CPR + AED (every patrol car carries an AED)	original list (Google Maps)
Police	PI Feldkirch	police patrol car (with AED)	9.58806	47.23489	24 h	CPR + AED (every patrol car carries an AED)	Schillerstrasse 9, 6800 Feldkirch (verified by authors, 13 Oct 2026)
Police	PI Sonntag	police patrol car (with AED)	9.89740	47.23930	24 h	CPR + AED (every patrol car carries an AED)	original list (Google Maps)
Police	PI Au	police patrol	9.98208	47.32127	24 h	CPR + AED	original list (Google

		car (with AED)				(every patrol car carries an AED)	Maps)
Police	PI Rankweil	police patrol car (with AED)	9.64330	47.27130	24 h	CPR + AED (every patrol car carries an AED)	original list (Google Maps)
Police	PI Rankweil Außenstelle Sulz	police patrol car (with AED)	9.65210	47.28480	24 h	CPR + AED (every patrol car carries an AED)	original list (Google Maps)
Police	PI Kleinwalsertal	police patrol car (with AED)	10.18560	47.35750	24 h	CPR + AED (every patrol car carries an AED)	original list (Google Maps)
Police	PI Götzis	police patrol car (with AED)	9.64650	47.33310	24 h	CPR + AED (every patrol car carries an AED)	original list (Google Maps)
Police	PI Bezau	police patrol car (with AED)	9.90220	47.38510	24 h	CPR + AED (every patrol car carries an AED)	original list (Google Maps)
Police	PI Altach	police patrol car (with AED)	9.63750	47.34840	24 h	CPR + AED (every patrol car carries an AED)	original list (Google Maps)
Police	PI Hohenems	police patrol car (with AED)	9.68740	47.36210	24 h	CPR + AED (every patrol car carries an AED)	original list (Google Maps)
Police	PI Egg	police patrol car (with AED)	9.89530	47.43080	24 h	CPR + AED (every patrol car carries an AED)	original list (Google Maps)
Police	PI Hittisau	police patrol car (with AED)	9.96080	47.45660	24 h	CPR + AED (every patrol car carries an AED)	original list (Google Maps)
Police	PI Dornbirn	police patrol car (with AED)	9.74220	47.41410	24 h	CPR + AED (every patrol car carries an AED)	original list (Google Maps)
Police	PI Lustenau	police patrol car (with AED)	9.66310	47.42620	24 h	CPR + AED (every patrol car carries an AED)	original list (Google Maps)
Police	PI Höchst	police patrol car (with AED)	9.63608	47.46057	24 h	CPR + AED (every patrol car carries an AED)	original list (Google Maps)
Police	PI Wolfurt	police patrol car (with AED)	9.75480	47.47250	24 h	CPR + AED (every patrol car carries an AED)	original list (Google Maps)
Police	PI Lauterach	police patrol car (with AED)	9.73110	47.47640	24 h	CPR + AED (every patrol car carries an AED)	original list (Google Maps)
Police	PI Hard	police patrol car (with AED)	9.68960	47.48720	24 h	CPR + AED (every patrol car carries an AED)	original list (Google Maps)
Police	PI Bregenz	police patrol car (with AED)	9.74230	47.50280	24 h	CPR + AED (every patrol car carries an AED)	original list (Google Maps)
Police	PI Hörbranz	police patrol car (with AED)	9.75316	47.55611	24 h	CPR + AED (every patrol car carries an AED)	original list (Google Maps)
Fire	BARTHOLOMÄBERG	fire engine (type varies by station)	9.91005	47.09124	24 h (volunteer, alerted)	CPR (BLS-trained); AED on fire vehicles neither mandatory nor standardised	Landesfeuerwehrverband Vorarlberg register
Fire	DORNBIRN	fire engine	9.74540	47.42227	24 h	CPR (BLS-	Landesfeuerwehrverband

		(type varies by station)			(volunteer, alerted)	trained); AED on fire vehicles neither mandatory nor standardised	Vorarlberg register
Fire	BARTHOLOMÄBERG	fire engine (type varies by station)	9.94373	47.10008	24 h (volunteer, alerted)	CPR (BLS-trained); AED on fire vehicles neither mandatory nor standardised	Landesfeuerwehrverband Vorarlberg register
Fire	ST.GEROLD	fire engine (type varies by station)	9.81755	47.22087	24 h (volunteer, alerted)	CPR (BLS-trained); AED on fire vehicles neither mandatory nor standardised	Landesfeuerwehrverband Vorarlberg register
Fire	BREGENZ	fire engine (type varies by station)	9.74888	47.50238	24 h (volunteer, alerted)	CPR (BLS-trained); AED on fire vehicles neither mandatory nor standardised	Landesfeuerwehrverband Vorarlberg register
Fire	ST.GALLENKIRCH	fire engine (type varies by station)	9.99893	47.00986	24 h (volunteer, alerted)	CPR (BLS-trained); AED on fire vehicles neither mandatory nor standardised	Landesfeuerwehrverband Vorarlberg register
Fire	ST.GALLENKIRCH	fire engine (type varies by station)	9.97060	47.02132	24 h (volunteer, alerted)	CPR (BLS-trained); AED on fire vehicles neither mandatory nor standardised	Landesfeuerwehrverband Vorarlberg register
Fire	BREGENZ	fire engine (type varies by station)	9.77495	47.49345	24 h (volunteer, alerted)	CPR (BLS-trained); AED on fire vehicles neither mandatory nor standardised	Landesfeuerwehrverband Vorarlberg register
Fire	THÜRINGERBERG	fire engine (type varies by station)	9.78504	47.21561	24 h (volunteer, alerted)	CPR (BLS-trained); AED on fire vehicles neither mandatory nor standardised	Landesfeuerwehrverband Vorarlberg register
Fire	SILBERTAL	fire engine (type varies by station)	9.98222	47.09371	24 h (volunteer, alerted)	CPR (BLS-trained); AED on fire vehicles neither mandatory nor standardised	Landesfeuerwehrverband Vorarlberg register
Fire	SONNTAG	fire engine (type varies by station)	9.90280	47.23920	24 h (volunteer, alerted)	CPR (BLS-trained); AED on fire vehicles neither mandatory nor standardised	Landesfeuerwehrverband Vorarlberg register
Fire	BÜRS	fire engine (type varies by station)	9.80108	47.15007	24 h (volunteer, alerted)	CPR (BLS-trained); AED on fire vehicles neither mandatory nor standardised	Landesfeuerwehrverband Vorarlberg register
Fire	DAMÜLS	fire engine (type varies by station)	9.88992	47.28198	24 h (volunteer, alerted)	CPR (BLS-trained); AED on fire vehicles neither mandatory nor standardised	Landesfeuerwehrverband Vorarlberg register
Fire	FELDKIRCH	fire engine (type varies by station)	9.58322	47.22628	24 h (volunteer, alerted)	CPR (BLS-trained); AED on fire vehicles neither mandatory nor standardised	Landesfeuerwehrverband Vorarlberg register
Fire	FELDKIRCH	fire engine (type varies	9.59220	47.23493	24 h (volunteer,	CPR (BLS-trained); AED	Landesfeuerwehrverband Vorarlberg register

		by station)			alerted)	on fire vehicles neither mandatory nor standardised	
Fire	DÜNSERBERG	fire engine (type varies by station)	9.72607	47.22638	24 h (volunteer, alerted)	CPR (BLS-trained); AED on fire vehicles neither mandatory nor standardised	Landesfeuerwehrverband Vorarlberg register
Fire	FELDKIRCH	fire engine (type varies by station)	9.62064	47.26105	24 h (volunteer, alerted)	CPR (BLS-trained); AED on fire vehicles neither mandatory nor standardised	Landesfeuerwehrverband Vorarlberg register
Fire	FELDKIRCH	fire engine (type varies by station)	9.59795	47.25954	24 h (volunteer, alerted)	CPR (BLS-trained); AED on fire vehicles neither mandatory nor standardised	Landesfeuerwehrverband Vorarlberg register
Fire	MEININGEN	fire engine (type varies by station)	9.57992	47.29838	24 h (volunteer, alerted)	CPR (BLS-trained); AED on fire vehicles neither mandatory nor standardised	Landesfeuerwehrverband Vorarlberg register
Fire	GASCHURN	fire engine (type varies by station)	10.02812	46.98318	24 h (volunteer, alerted)	CPR (BLS-trained); AED on fire vehicles neither mandatory nor standardised	Landesfeuerwehrverband Vorarlberg register
Fire	LECH	fire engine (type varies by station)	10.15187	47.21810	24 h (volunteer, alerted)	CPR (BLS-trained); AED on fire vehicles neither mandatory nor standardised	Landesfeuerwehrverband Vorarlberg register
Fire	ST.ANTON	fire engine (type varies by station)	9.86523	47.11288	24 h (volunteer, alerted)	CPR (BLS-trained); AED on fire vehicles neither mandatory nor standardised	Landesfeuerwehrverband Vorarlberg register
Fire	BLUDESCH	fire engine (type varies by station)	9.74500	47.19921	24 h (volunteer, alerted)	CPR (BLS-trained); AED on fire vehicles neither mandatory nor standardised	Landesfeuerwehrverband Vorarlberg register
Fire	MITTELBERG	fire engine (type varies by station)	10.15331	47.32308	24 h (volunteer, alerted)	CPR (BLS-trained); AED on fire vehicles neither mandatory nor standardised	Landesfeuerwehrverband Vorarlberg register
Fire	SCHNIFIS	fire engine (type varies by station)	9.72880	47.21372	24 h (volunteer, alerted)	CPR (BLS-trained); AED on fire vehicles neither mandatory nor standardised	Landesfeuerwehrverband Vorarlberg register
Fire	HOHENEMS	fire engine (type varies by station)	9.67986	47.36225	24 h (volunteer, alerted)	CPR (BLS-trained); AED on fire vehicles neither mandatory nor standardised	Landesfeuerwehrverband Vorarlberg register
Fire	ANDELSBUCH	fire engine (type varies by station)	9.89208	47.41090	24 h (volunteer, alerted)	CPR (BLS-trained); AED on fire vehicles neither mandatory nor standardised	Landesfeuerwehrverband Vorarlberg register
Fire	BEZAU	fire engine (type varies by station)	9.90071	47.38445	24 h (volunteer, alerted)	CPR (BLS-trained); AED on fire vehicles	Landesfeuerwehrverband Vorarlberg register

						neither mandatory nor standardised	
Fire	BILDSTEIN	fire engine (type varies by station)	9.77404	47.45802	24 h (volunteer, alerted)	CPR (BLS-trained); AED on fire vehicles neither mandatory nor standardised	Landesfeuerwehrverband Vorarlberg register
Fire	BIZAU	fire engine (type varies by station)	9.93182	47.37002	24 h (volunteer, alerted)	CPR (BLS-trained); AED on fire vehicles neither mandatory nor standardised	Landesfeuerwehrverband Vorarlberg register
Fire	BLONS	fire engine (type varies by station)	9.83846	47.22443	24 h (volunteer, alerted)	CPR (BLS-trained); AED on fire vehicles neither mandatory nor standardised	Landesfeuerwehrverband Vorarlberg register
Fire	ZWISCHENWASSER	fire engine (type varies by station)	9.68722	47.29399	24 h (volunteer, alerted)	CPR (BLS-trained); AED on fire vehicles neither mandatory nor standardised	Landesfeuerwehrverband Vorarlberg register
Fire	BRAND	fire engine (type varies by station)	9.73819	47.10332	24 h (volunteer, alerted)	CPR (BLS-trained); AED on fire vehicles neither mandatory nor standardised	Landesfeuerwehrverband Vorarlberg register
Fire	MÖGGERS	fire engine (type varies by station)	9.81759	47.56200	24 h (volunteer, alerted)	CPR (BLS-trained); AED on fire vehicles neither mandatory nor standardised	Landesfeuerwehrverband Vorarlberg register
Fire	REUTHE	fire engine (type varies by station)	9.88690	47.37915	24 h (volunteer, alerted)	CPR (BLS-trained); AED on fire vehicles neither mandatory nor standardised	Landesfeuerwehrverband Vorarlberg register
Fire	RIEFENSBERG	fire engine (type varies by station)	9.95932	47.50132	24 h (volunteer, alerted)	CPR (BLS-trained); AED on fire vehicles neither mandatory nor standardised	Landesfeuerwehrverband Vorarlberg register
Fire	SATTEINS	fire engine (type varies by station)	9.67376	47.22640	24 h (volunteer, alerted)	CPR (BLS-trained); AED on fire vehicles neither mandatory nor standardised	Landesfeuerwehrverband Vorarlberg register
Fire	RÖTHIS	fire engine (type varies by station)	9.65157	47.29160	24 h (volunteer, alerted)	CPR (BLS-trained); AED on fire vehicles neither mandatory nor standardised	Landesfeuerwehrverband Vorarlberg register
Fire	BREGENZ	fire engine (type varies by station)	9.73024	47.49077	24 h (volunteer, alerted)	CPR (BLS-trained); AED on fire vehicles neither mandatory nor standardised	Landesfeuerwehrverband Vorarlberg register
Fire	BREGENZ	fire engine (type varies by station)	9.71989	47.49457	24 h (volunteer, alerted)	CPR (BLS-trained); AED on fire vehicles neither mandatory nor standardised	Landesfeuerwehrverband Vorarlberg register
Fire	SCHNEPFAU	fire engine (type varies by station)	9.94519	47.35226	24 h (volunteer, alerted)	CPR (BLS-trained); AED on fire vehicles neither	Landesfeuerwehrverband Vorarlberg register

						mandatory nor standardised	
Fire	SCHWARZACH	fire engine (type varies by station)	9.76210	47.44687	24 h (volunteer, alerted)	CPR (BLS-trained); AED on fire vehicles neither mandatory nor standardised	Landesfeuerwehrverband Vorarlberg register
Fire	SULZBERG	fire engine (type varies by station)	9.91428	47.52223	24 h (volunteer, alerted)	CPR (BLS-trained); AED on fire vehicles neither mandatory nor standardised	Landesfeuerwehrverband Vorarlberg register
Fire	SULZ	fire engine (type varies by station)	9.65284	47.28851	24 h (volunteer, alerted)	CPR (BLS-trained); AED on fire vehicles neither mandatory nor standardised	Landesfeuerwehrverband Vorarlberg register
Fire	ÜBERSAXEN	fire engine (type varies by station)	9.67317	47.25251	24 h (volunteer, alerted)	CPR (BLS-trained); AED on fire vehicles neither mandatory nor standardised	Landesfeuerwehrverband Vorarlberg register
Fire	WEILER	fire engine (type varies by station)	9.65344	47.30388	24 h (volunteer, alerted)	CPR (BLS-trained); AED on fire vehicles neither mandatory nor standardised	Landesfeuerwehrverband Vorarlberg register
Fire	ZWISCHENWASSER	fire engine (type varies by station)	9.66349	47.28820	24 h (volunteer, alerted)	CPR (BLS-trained); AED on fire vehicles neither mandatory nor standardised	Landesfeuerwehrverband Vorarlberg register
Fire	THÜRINGEN	fire engine (type varies by station)	9.76965	47.19808	24 h (volunteer, alerted)	CPR (BLS-trained); AED on fire vehicles neither mandatory nor standardised	Landesfeuerwehrverband Vorarlberg register
Fire	DOREN	fire engine (type varies by station)	9.89047	47.49493	24 h (volunteer, alerted)	CPR (BLS-trained); AED on fire vehicles neither mandatory nor standardised	Landesfeuerwehrverband Vorarlberg register
Fire	LORÜNS	fire engine (type varies by station)	9.85281	47.12969	24 h (volunteer, alerted)	CPR (BLS-trained); AED on fire vehicles neither mandatory nor standardised	Landesfeuerwehrverband Vorarlberg register
Fire	LUDESCH	fire engine (type varies by station)	9.78266	47.19305	24 h (volunteer, alerted)	CPR (BLS-trained); AED on fire vehicles neither mandatory nor standardised	Landesfeuerwehrverband Vorarlberg register
Fire	LUSTENAU	fire engine (type varies by station)	9.66321	47.43294	24 h (volunteer, alerted)	CPR (BLS-trained); AED on fire vehicles neither mandatory nor standardised	Landesfeuerwehrverband Vorarlberg register
Fire	KENNELBACH	fire engine (type varies by station)	9.76144	47.47852	24 h (volunteer, alerted)	CPR (BLS-trained); AED on fire vehicles neither mandatory nor standardised	Landesfeuerwehrverband Vorarlberg register
Fire	EICHENBERG	fire engine (type varies by station)	9.77981	47.53267	24 h (volunteer, alerted)	CPR (BLS-trained); AED on fire vehicles neither mandatory nor	Landesfeuerwehrverband Vorarlberg register

						standardised	
Fire	AU	fire engine (type varies by station)	9.98336	47.32281	24 h (volunteer, alerted)	CPR (BLS-trained); AED on fire vehicles neither mandatory nor standardised	Landesfeuerwehrverband Vorarlberg register
Fire	FRASTANZ	fire engine (type varies by station)	9.63744	47.21945	24 h (volunteer, alerted)	CPR (BLS-trained); AED on fire vehicles neither mandatory nor standardised	Landesfeuerwehrverband Vorarlberg register
Fire	BLUDENZ	fire engine (type varies by station)	9.82120	47.15844	24 h (volunteer, alerted)	CPR (BLS-trained); AED on fire vehicles neither mandatory nor standardised	Landesfeuerwehrverband Vorarlberg register
Fire	ST.GALLENKIRCH	fire engine (type varies by station)	9.91824	46.97198	24 h (volunteer, alerted)	CPR (BLS-trained); AED on fire vehicles neither mandatory nor standardised	Landesfeuerwehrverband Vorarlberg register
Fire	EGG	fire engine (type varies by station)	9.89799	47.43282	24 h (volunteer, alerted)	CPR (BLS-trained); AED on fire vehicles neither mandatory nor standardised	Landesfeuerwehrverband Vorarlberg register
Fire	NÜZIDERS	fire engine (type varies by station)	9.79820	47.17081	24 h (volunteer, alerted)	CPR (BLS-trained); AED on fire vehicles neither mandatory nor standardised	Landesfeuerwehrverband Vorarlberg register
Fire	RAGGAL	fire engine (type varies by station)	9.83735	47.21121	24 h (volunteer, alerted)	CPR (BLS-trained); AED on fire vehicles neither mandatory nor standardised	Landesfeuerwehrverband Vorarlberg register
Fire	FELDKIRCH	fire engine (type varies by station)	9.57617	47.23880	24 h (volunteer, alerted)	CPR (BLS-trained); AED on fire vehicles neither mandatory nor standardised	Landesfeuerwehrverband Vorarlberg register
Fire	ALBERSCHWENDE	fire engine (type varies by station)	9.87725	47.45419	24 h (volunteer, alerted)	CPR (BLS-trained); AED on fire vehicles neither mandatory nor standardised	Landesfeuerwehrverband Vorarlberg register
Fire	WOLFURT	fire engine (type varies by station)	9.74575	47.46087	24 h (volunteer, alerted)	CPR (BLS-trained); AED on fire vehicles neither mandatory nor standardised	Landesfeuerwehrverband Vorarlberg register
Fire	LATERNS	fire engine (type varies by station)	9.73259	47.26227	24 h (volunteer, alerted)	CPR (BLS-trained); AED on fire vehicles neither mandatory nor standardised	Landesfeuerwehrverband Vorarlberg register
Fire	BÜRSERBERG	fire engine (type varies by station)	9.77769	47.14215	24 h (volunteer, alerted)	CPR (BLS-trained); AED on fire vehicles neither mandatory nor standardised	Landesfeuerwehrverband Vorarlberg register
Fire	FUßACH	fire engine (type varies by station)	9.66232	47.47407	24 h (volunteer, alerted)	CPR (BLS-trained); AED on fire vehicles neither mandatory nor standardised	Landesfeuerwehrverband Vorarlberg register

Fire	SCHOPPERNAU	fire engine (type varies by station)	10.01596	47.31192	24 h (volunteer, alerted)	CPR (BLS-trained); AED on fire vehicles neither mandatory nor standardised	Landesfeuerwehrverband Vorarlberg register
Fire	KLÖSTERLE	fire engine (type varies by station)	10.09221	47.13140	24 h (volunteer, alerted)	CPR (BLS-trained); AED on fire vehicles neither mandatory nor standardised	Landesfeuerwehrverband Vorarlberg register
Fire	EGG	fire engine (type varies by station)	9.91415	47.43443	24 h (volunteer, alerted)	CPR (BLS-trained); AED on fire vehicles neither mandatory nor standardised	Landesfeuerwehrverband Vorarlberg register
Fire	GAIßAU	fire engine (type varies by station)	9.59647	47.46647	24 h (volunteer, alerted)	CPR (BLS-trained); AED on fire vehicles neither mandatory nor standardised	Landesfeuerwehrverband Vorarlberg register
Fire	HARD	fire engine (type varies by station)	9.69061	47.49170	24 h (volunteer, alerted)	CPR (BLS-trained); AED on fire vehicles neither mandatory nor standardised	Landesfeuerwehrverband Vorarlberg register
Fire	NENZING	fire engine (type varies by station)	9.64654	47.20890	24 h (volunteer, alerted)	CPR (BLS-trained); AED on fire vehicles neither mandatory nor standardised	Landesfeuerwehrverband Vorarlberg register
Fire	KLÖSTERLE	fire engine (type varies by station)	10.15836	47.13814	24 h (volunteer, alerted)	CPR (BLS-trained); AED on fire vehicles neither mandatory nor standardised	Landesfeuerwehrverband Vorarlberg register
Fire	GÖTZIS	fire engine (type varies by station)	9.63278	47.33072	24 h (volunteer, alerted)	CPR (BLS-trained); AED on fire vehicles neither mandatory nor standardised	Landesfeuerwehrverband Vorarlberg register
Fire	HOHENEMS	fire engine (type varies by station)	9.71458	47.37281	24 h (volunteer, alerted)	CPR (BLS-trained); AED on fire vehicles neither mandatory nor standardised	Landesfeuerwehrverband Vorarlberg register
Fire	SCHRUNS	fire engine (type varies by station)	9.92038	47.07843	24 h (volunteer, alerted)	CPR (BLS-trained); AED on fire vehicles neither mandatory nor standardised	Landesfeuerwehrverband Vorarlberg register
Fire	TSCHAGGUNS	fire engine (type varies by station)	9.87643	47.07329	24 h (volunteer, alerted)	CPR (BLS-trained); AED on fire vehicles neither mandatory nor standardised	Landesfeuerwehrverband Vorarlberg register
Fire	TSCHAGGUNS	fire engine (type varies by station)	9.90353	47.07584	24 h (volunteer, alerted)	CPR (BLS-trained); AED on fire vehicles neither mandatory nor standardised	Landesfeuerwehrverband Vorarlberg register
Fire	FELDKIRCH	fire engine (type varies by station)	9.57865	47.25665	24 h (volunteer, alerted)	CPR (BLS-trained); AED on fire vehicles neither mandatory nor standardised	Landesfeuerwehrverband Vorarlberg register
Fire	SCHWARZENBERG	fire engine	9.85110	47.41624	24 h	CPR (BLS-	Landesfeuerwehrverband

		(type varies by station)			(volunteer, alerted)	trained); AED on fire vehicles neither mandatory nor standardised	Vorarlberg register
Fire	VIKTORSBERG	fire engine (type varies by station)	9.67502	47.30175	24 h (volunteer, alerted)	CPR (BLS-trained); AED on fire vehicles neither mandatory nor standardised	Landesfeuerwehrverband Vorarlberg register
Fire	ALBERSCHWENDE	fire engine (type varies by station)	9.82765	47.45109	24 h (volunteer, alerted)	CPR (BLS-trained); AED on fire vehicles neither mandatory nor standardised	Landesfeuerwehrverband Vorarlberg register
Fire	KOBLACH	fire engine (type varies by station)	9.59854	47.33135	24 h (volunteer, alerted)	CPR (BLS-trained); AED on fire vehicles neither mandatory nor standardised	Landesfeuerwehrverband Vorarlberg register
Fire	DÜNS	fire engine (type varies by station)	9.72049	47.22077	24 h (volunteer, alerted)	CPR (BLS-trained); AED on fire vehicles neither mandatory nor standardised	Landesfeuerwehrverband Vorarlberg register
Fire	BLUDENZ	fire engine (type varies by station)	9.85526	47.14224	24 h (volunteer, alerted)	CPR (BLS-trained); AED on fire vehicles neither mandatory nor standardised	Landesfeuerwehrverband Vorarlberg register
Fire	VANDANS	fire engine (type varies by station)	9.86342	47.09582	24 h (volunteer, alerted)	CPR (BLS-trained); AED on fire vehicles neither mandatory nor standardised	Landesfeuerwehrverband Vorarlberg register
Fire	LECH	fire engine (type varies by station)	10.16460	47.16951	24 h (volunteer, alerted)	CPR (BLS-trained); AED on fire vehicles neither mandatory nor standardised	Landesfeuerwehrverband Vorarlberg register
Fire	GÖFIS	fire engine (type varies by station)	9.63738	47.23738	24 h (volunteer, alerted)	CPR (BLS-trained); AED on fire vehicles neither mandatory nor standardised	Landesfeuerwehrverband Vorarlberg register
Fire	SULZBERG	fire engine (type varies by station)	9.88234	47.53693	24 h (volunteer, alerted)	CPR (BLS-trained); AED on fire vehicles neither mandatory nor standardised	Landesfeuerwehrverband Vorarlberg register
Fire	BUCH	fire engine (type varies by station)	9.81858	47.48209	24 h (volunteer, alerted)	CPR (BLS-trained); AED on fire vehicles neither mandatory nor standardised	Landesfeuerwehrverband Vorarlberg register
Fire	NENZING	fire engine (type varies by station)	9.64158	47.19372	24 h (volunteer, alerted)	CPR (BLS-trained); AED on fire vehicles neither mandatory nor standardised	Landesfeuerwehrverband Vorarlberg register
Fire	HOHENWEILER	fire engine (type varies by station)	9.78080	47.58685	24 h (volunteer, alerted)	CPR (BLS-trained); AED on fire vehicles neither mandatory nor standardised	Landesfeuerwehrverband Vorarlberg register
Fire	LANGEN	fire engine (type varies	9.82902	47.50796	24 h (volunteer,	CPR (BLS-trained); AED	Landesfeuerwehrverband Vorarlberg register

		by station)			alerted)	on fire vehicles neither mandatory nor standardised	
Fire	LANGENEGG	fire engine (type varies by station)	9.89690	47.46860	24 h (volunteer, alerted)	CPR (BLS-trained); AED on fire vehicles neither mandatory nor standardised	Landesfeuerwehrverband Vorarlberg register
Fire	KLAUS	fire engine (type varies by station)	9.64561	47.30825	24 h (volunteer, alerted)	CPR (BLS-trained); AED on fire vehicles neither mandatory nor standardised	Landesfeuerwehrverband Vorarlberg register
Fire	SCHLINS	fire engine (type varies by station)	9.69354	47.20191	24 h (volunteer, alerted)	CPR (BLS-trained); AED on fire vehicles neither mandatory nor standardised	Landesfeuerwehrverband Vorarlberg register
Fire	FONTANELLA	fire engine (type varies by station)	9.91118	47.24801	24 h (volunteer, alerted)	CPR (BLS-trained); AED on fire vehicles neither mandatory nor standardised	Landesfeuerwehrverband Vorarlberg register
Fire	INNERBRAZ	fire engine (type varies by station)	9.92296	47.14272	24 h (volunteer, alerted)	CPR (BLS-trained); AED on fire vehicles neither mandatory nor standardised	Landesfeuerwehrverband Vorarlberg register
Fire	LAUTERACH	fire engine (type varies by station)	9.72724	47.47673	24 h (volunteer, alerted)	CPR (BLS-trained); AED on fire vehicles neither mandatory nor standardised	Landesfeuerwehrverband Vorarlberg register
Fire	LINGENAU	fire engine (type varies by station)	9.91989	47.45156	24 h (volunteer, alerted)	CPR (BLS-trained); AED on fire vehicles neither mandatory nor standardised	Landesfeuerwehrverband Vorarlberg register
Fire	LOCHAU	fire engine (type varies by station)	9.75192	47.53431	24 h (volunteer, alerted)	CPR (BLS-trained); AED on fire vehicles neither mandatory nor standardised	Landesfeuerwehrverband Vorarlberg register
Fire	MÄDER	fire engine (type varies by station)	9.61761	47.35028	24 h (volunteer, alerted)	CPR (BLS-trained); AED on fire vehicles neither mandatory nor standardised	Landesfeuerwehrverband Vorarlberg register
Fire	RANKWEIL	fire engine (type varies by station)	9.64266	47.27304	24 h (volunteer, alerted)	CPR (BLS-trained); AED on fire vehicles neither mandatory nor standardised	Landesfeuerwehrverband Vorarlberg register
Fire	DORNBIRN	fire engine (type varies by station)	9.75126	47.35073	24 h (volunteer, alerted)	CPR (BLS-trained); AED on fire vehicles neither mandatory nor standardised	Landesfeuerwehrverband Vorarlberg register
Fire	WARTH	fire engine (type varies by station)	10.18587	47.25902	24 h (volunteer, alerted)	CPR (BLS-trained); AED on fire vehicles neither mandatory nor standardised	Landesfeuerwehrverband Vorarlberg register
Fire	SCHRÖCKEN	fire engine (type varies by station)	10.09295	47.25761	24 h (volunteer, alerted)	CPR (BLS-trained); AED on fire vehicles	Landesfeuerwehrverband Vorarlberg register

						neither mandatory nor standardised	
Fire	ZWISCHENWASSER	fire engine (type varies by station)	9.66635	47.27848	24 h (volunteer, alerted)	CPR (BLS-trained); AED on fire vehicles neither mandatory nor standardised	Landesfeuerwehrverband Vorarlberg register
Fire	GASCHURN	fire engine (type varies by station)	10.06023	46.96847	24 h (volunteer, alerted)	CPR (BLS-trained); AED on fire vehicles neither mandatory nor standardised	Landesfeuerwehrverband Vorarlberg register
Fire	KRUMBACH	fire engine (type varies by station)	9.93558	47.48349	24 h (volunteer, alerted)	CPR (BLS-trained); AED on fire vehicles neither mandatory nor standardised	Landesfeuerwehrverband Vorarlberg register
Fire	MELLAU	fire engine (type varies by station)	9.87952	47.34878	24 h (volunteer, alerted)	CPR (BLS-trained); AED on fire vehicles neither mandatory nor standardised	Landesfeuerwehrverband Vorarlberg register
Fire	RÖNS	fire engine (type varies by station)	9.69886	47.21647	24 h (volunteer, alerted)	CPR (BLS-trained); AED on fire vehicles neither mandatory nor standardised	Landesfeuerwehrverband Vorarlberg register
Fire	MITTELBERG	fire engine (type varies by station)	10.16731	47.34254	24 h (volunteer, alerted)	CPR (BLS-trained); AED on fire vehicles neither mandatory nor standardised	Landesfeuerwehrverband Vorarlberg register
Fire	DALAAS	fire engine (type varies by station)	10.04382	47.13082	24 h (volunteer, alerted)	CPR (BLS-trained); AED on fire vehicles neither mandatory nor standardised	Landesfeuerwehrverband Vorarlberg register
Fire	HÖRBRANZ	fire engine (type varies by station)	9.75126	47.55881	24 h (volunteer, alerted)	CPR (BLS-trained); AED on fire vehicles neither mandatory nor standardised	Landesfeuerwehrverband Vorarlberg register
Fire	DALAAS	fire engine (type varies by station)	9.99708	47.12213	24 h (volunteer, alerted)	CPR (BLS-trained); AED on fire vehicles neither mandatory nor standardised	Landesfeuerwehrverband Vorarlberg register
Fire	MITTELBERG	fire engine (type varies by station)	10.18597	47.35765	24 h (volunteer, alerted)	CPR (BLS-trained); AED on fire vehicles neither mandatory nor standardised	Landesfeuerwehrverband Vorarlberg register
Fire	HITTISAU	fire engine (type varies by station)	9.96126	47.45638	24 h (volunteer, alerted)	CPR (BLS-trained); AED on fire vehicles neither mandatory nor standardised	Landesfeuerwehrverband Vorarlberg register
Fire	SIBRATSGFÄLL	fire engine (type varies by station)	10.03519	47.43000	24 h (volunteer, alerted)	CPR (BLS-trained); AED on fire vehicles neither mandatory nor standardised	Landesfeuerwehrverband Vorarlberg register
Fire	ALTACH	fire engine (type varies by station)	9.64824	47.35874	24 h (volunteer, alerted)	CPR (BLS-trained); AED on fire vehicles neither	Landesfeuerwehrverband Vorarlberg register

						mandatory nor standardised	
Fire	HÖCHST	fire engine (type varies by station)	9.64247	47.45739	24 h (volunteer, alerted)	CPR (BLS-trained); AED on fire vehicles neither mandatory nor standardised	Landesfeuerwehrverband Vorarlberg register

Table S4a. Monte Carlo uncertainty: bootstrap confidence intervals. Point estimates with 95% paired bootstrap confidence intervals (2000 resamples, seed 20262012).

Estimate	Point estimate	95% CI, lower	95% CI, upper
Median response time, EMS alone (min)	9.81	9.29	10.67
Coverage ≤10 min, EMS alone (%)	51.10	46.69	55.31
Median response time, EMS + police (min)	5.45	5.12	5.79
Coverage ≤10 min, EMS + police (%)	87.37	84.37	89.98
Median paired time gain, EMS + police minus EMS alone (min, positive = faster)	3.79	3.26	4.29
Coverage gain, EMS + police minus EMS alone (pp)	36.27	32.26	40.29
Median response time, EMS + fire brigade (min)	7.36	7.11	7.61
Coverage ≤10 min, EMS + fire brigade (%)	86.97	83.97	89.98
Median paired time gain, EMS + fire brigade minus EMS alone (min, positive = faster)	1.21	0.68	1.83
Coverage gain, EMS + fire brigade minus EMS alone (pp)	35.87	31.86	40.08
Median response time, triple dispatch (min)	5.41	5.11	5.62
Coverage ≤10 min, triple dispatch (%)	94.59	92.59	96.59
Median paired time gain, triple dispatch minus EMS alone (min, positive = faster)	4.34	3.80	4.67
Coverage gain, triple dispatch minus EMS alone (pp)	43.49	39.28	47.90
Coverage difference, EMS + police minus EMS + fire brigade (pp)	0.40	-2.81	3.81
Median paired difference in response time, EMS + police minus EMS + fire brigade (min, negative = police faster)	-1.59	-2.06	-1.14
Coverage gain, triple dispatch minus EMS + police (pp)	7.21	5.01	9.62
Mean paired time gain, triple dispatch vs. EMS + police (min)	0.59	0.43	0.77
Coverage gain, triple dispatch minus EMS + fire brigade (pp)	7.62	5.41	10.02
Mean paired time gain, triple dispatch vs. EMS + fire brigade (min)	2.12	1.93	2.30

Table S4b. Monte Carlo uncertainty: replicate samples. Variation across 20 independent samples of 500 locations (seeds 20261014–20261033).

Estimate	Mean across samples	SD between samples	2.5th percentile	97.5th percentile
Median response time, EMS alone (min)	10.03	0.43	9.40	10.77
Coverage ≤10 min, EMS alone (%)	49.88	2.44	45.90	54.29
Median response time, EMS + police (min)	5.32	0.18	4.94	5.60
Coverage ≤10 min, EMS + police (%)	88.62	1.78	85.53	91.88

Median paired time gain, EMS + police minus EMS alone (min, positive = faster)	4.01	0.30	3.51	4.50
Coverage gain, EMS + police minus EMS alone (pp)	38.74	1.84	35.69	41.81
Median response time, EMS + fire brigade (min)	7.28	0.15	7.04	7.56
Coverage ≤10 min, EMS + fire brigade (%)	87.81	1.98	83.84	90.38
Median paired time gain, EMS + fire brigade minus EMS alone (min, positive = faster)	1.28	0.23	0.82	1.75
Coverage gain, EMS + fire brigade minus EMS alone (pp)	37.93	2.05	33.61	41.76
Median response time, triple dispatch (min)	5.29	0.17	4.94	5.52
Coverage ≤10 min, triple dispatch (%)	94.74	1.15	92.50	96.69
Median paired time gain, triple dispatch minus EMS alone (min, positive = faster)	4.48	0.24	4.07	4.70
Coverage gain, triple dispatch minus EMS alone (pp)	44.85	2.21	40.79	48.62
Coverage difference, EMS + police minus EMS + fire brigade (pp)	0.81	1.43	-1.41	3.21
Median paired difference in response time, EMS + police minus EMS + fire brigade (min, negative = police faster)	-1.58	0.20	-1.91	-1.24
Coverage gain, triple dispatch minus EMS + police (pp)	6.12	1.00	4.51	8.02
Mean paired time gain, triple dispatch vs. EMS + police (min)	0.56	0.08	0.45	0.71
Coverage gain, triple dispatch minus EMS + fire brigade (pp)	6.93	1.38	4.70	9.23
Mean paired time gain, triple dispatch vs. EMS + fire brigade (min)	2.12	0.11	1.93	2.29

Table S4c. Monte Carlo uncertainty: population epochs. Results for seeded samples drawn from each GHS-POP epoch.

**GHS-POP epoch**	**Configuration**	**Median (min)**	**Q1 (min)**	**Q3 (min)**	**Coverage ≤10 min (%)**	**Median paired gain vs. EMS alone (min)**	**Coverage gain vs. EMS alone (pp)**
E2030	EMS alone	9.22	6.88	14.49	54.00	–	–
E2030	EMS + police	5.45	3.57	7.99	86.80	3.47	32.80
E2030	EMS + fire brigade	7.31	6.03	8.70	86.60	0.55	32.60
E2030	triple dispatch	5.41	3.57	7.33	93.80	3.96	39.80
E2025	EMS alone	9.90	6.88	14.80	50.50	–	–
E2025	EMS + police	5.26	3.41	7.95	86.17	3.62	35.67

E2025	EMS + fire brigade	7.38	6.20	8.73	85.97	0.73	35.47
E2025	triple dispatch	5.26	3.41	7.08	93.99	4.51	43.49
E2020	EMS alone	9.47	6.74	14.69	53.40	–	–
E2020	EMS + police	5.37	3.56	7.86	86.60	3.46	33.20
E2020	EMS + fire brigade	7.45	5.92	8.81	88.00	1.07	34.60
E2020	triple dispatch	5.32	3.56	7.16	95.20	4.10	41.80

Table S5. Unit availability, stochastic turnout and travel-time scenarios. Coverage (≤10 min, %) under reduced unit availability, night-time closure of day-only EMS stations, stochastic turnout times (EMS uniform 1–3 min, police uniform 0–3 min, fire brigade log-normal with median 5 and 90th percentile 9 min) and travel-time multipliers; mean of 500 Monte Carlo replications (2.5th–97.5th percentiles are reproduced by Code S1).

Scenario	EMS alone (%)	EMS + police (%)	EMS + fire brigade (%)	triple dispatch (%)
Availability high	48.10	83.30	85.00	93.20
Availability low	40.80	68.80	78.30	87.00
Availability moderate	44.70	77.80	82.70	91.20
Combined moderate	41.50	70.50	79.80	87.60
Deterministic base case	51.10	87.40	87.00	94.60
Night: day-only EMS closed	48.70	86.40	86.80	94.60
Stochastic turnout only	50.70	81.90	84.40	91.50
Travel time x 0.85 (emergency driving)	58.90	92.60	91.60	95.60
Travel time x 1.20 (winter/congestion)	41.50	79.20	78.60	90.40

Table S6. Results by district, degree of urbanisation and EMS response stratum. Coverage (≤10 min, %) and median paired time gain; degree of urbanisation from GHS-SMOD.

Stratifier	Stratum	Locations (n)	Coverage ≤10 min, EMS alone (%)	Coverage ≤10 min, EMS + police (%)	Coverage ≤10 min, EMS + fire brigade (%)	Coverage ≤10 min, triple dispatch (%)	Median paired gain, EMS + police minus EMS alone (min)	Median paired gain, EMS + fire brigade minus EMS alone (min)	Median paired gain, triple dispatch minus EMS alone (min)
District	Bludenz	69	31.90	73.90	75.40	84.10	4.80	4.50	5.50
District	Bregenz	184	47.30	86.40	86.40	95.10	4.10	1.80	5.00
District	Dornbirn	111	89.20	98.20	93.70	98.20	2.50	0.00	2.50
District	Feldkirch	135	34.80	86.70	88.10	96.30	5.80	3.70	5.80
Degree of urbanisation	Rural	133	16.50	55.60	70.70	79.70	4.10	5.20	6.60
Degree of urbanisation	Urban cluster	366	63.70	98.90	92.90	100	3.60	0.20	3.60
EMS alone	EMS >10 min	244	0.00	74.20	73.40	88.90	8.10	6.00	8.90
EMS alone	EMS ≤10 min	255	100	100	100	100	2.00	0.00	2.10

Table S7a. Decomposition of the difference between isochrone- and mission-based coverage. Mission-based coverage: share of the 499 simulated locations reached; isochrone-based coverage: share of residents inside the isochrones; column 'basis' gives the denominator of each row. Each step changes one element relative to the previous one. A full-grid step (all populated cells instead of a sample) was not run because of the routing quota; sampling variation is quantified in Table S4 and, for the same locations, in Table S7b. Setting the isochrone smoothing to 0 did not change the polygons.

No.	Step	EMS alone (%)	EMS + police (%)	EMS + fire brigade (%)	triple dispatch (%)	Denominator
1	Published analysis: original routing (incident->station, 10 nearest stations by straight line), <10 min	50.90	86.20	87.40	94.40	% of simulated locations
2	All stations routed, incident->station, <10 min	51.10	87.40	87.40	94.60	% of simulated locations
3	All stations, station->incident, <10 min	51.10	87.40	87.00	94.60	% of simulated locations
4	All stations, station->incident, <=10 min (revised main analysis)	51.10	87.40	87.00	94.60	% of simulated locations
5	Isochrones, default smoothing, population by cell centre	55.60	90.90	91.90	95.50	% of residents
6	Isochrones, default smoothing, population of all touched cells	57.00	91.60	93.20	96.00	% of residents
7	Isochrones, smoothing = 0 (minimal interpolation)	55.60	90.90	91.90	95.50	% of residents

Table S7b. Same-point comparison of isochrones and matrix travel times. Share of the simulated locations inside each isochrone and reached within the threshold in the matrix analysis; discordant locations with their median response time and snapping distance.

Configuration	Locations (n)	Residents inside isochrone (%)	Locations inside isochrone (%)	Locations reached ≤10 min, matrix (%)	Inside isochrone, not reached (n)	Reached, outside isochrone (n)	Median response time, inside but not reached (min)	Maximum response time, inside but not reached (min)	Median snapping distance, inside but not reached (m)	Median snapping distance, all locations (m)	Residents minus locations inside isochrone (pp)	Locations inside isochrone minus locations reached (pp)
EMS alone	499	55.63	55.31	51.10	22	1	10.60	12.22	11.10	14.31	0.32	4.21
EMS + police	499	90.94	89.58	87.37	12	1	10.45	14.23	22.27	14.31	1.36	2.20
EMS + fire brigade	499	91.89	90.38	86.97	21	4	10.68	12.52	12.76	14.31	1.51	3.41

triple dispatch	499	95.50	94.59	94.59	3	3	11.28	14.23	21.04	14.31	0.91	0.00

Table S8. Comparison of the dual configurations. Head-to-head comparison of EMS + police and EMS + fire brigade; differences are given as EMS + police minus EMS + fire brigade.

**Measure**	**Value**
Comparison	EMS + Police vs. EMS + Fire
Locations (n)	499
EMS + police faster (% of locations)	69.1
EMS + fire brigade faster (% of locations)	20.0
Tie (% of locations)	10.8
Median paired difference in response time, police minus fire (min)	-1.59
Hodges-Lehmann estimate, police minus fire (min)	-1.71
Sign test, p (ties excluded)	<0.001
Wilcoxon signed-rank test, p (ties excluded)	<0.001
Wilcoxon signed-rank test, p (Pratt)	<0.001
Wilcoxon signed-rank test, p (zero-splitting)	<0.001
Matched-pairs rank-biserial correlation	-0.61
Coverage ≤10 min, EMS + police (%)	87.4
Coverage ≤10 min, EMS + fire brigade (%)	87.0
Coverage difference, police minus fire (pp)	0.4
Reached ≤10 min only with EMS + police (n)	38
Reached ≤10 min only with EMS + fire brigade (n)	36
Exact McNemar test, p	0.908

Table S9. Incremental value of triple dispatch over each dual configuration.

**Comparison**	**Median paired gain (min)**	**Q3 of paired gain (min)**	**90th percentile of paired gain (min)**	**Mean paired gain (min)**	**Triple dispatch faster (% of locations)**	**Median gain where faster (min)**	**Additional locations reached ≤10 min (n)**	**Coverage gain (pp)**
Triple vs. EMS + Police	0.00	0.00	1.79	0.59	20.04	1.79	36	7.21
Triple vs. EMS + Fire	1.59	3.72	5.48	2.12	69.14	2.73	38	7.62
